# Masticatory Dysfunction by Extensive Tooth Loss as a Risk Factor for Cognitive Deficit: A Systematic Review and Meta-Analysis

**DOI:** 10.3389/fphys.2019.00832

**Published:** 2019-07-03

**Authors:** María Olimpia Paz Alvarenga, Railson de Oliveira Ferreira, Marcela Baraúna Magno, Nathalia Carolina Fernandes Fagundes, Lucianne Cople Maia, Rafael Rodrigues Lima

**Affiliations:** ^1^Laboratory of Functional and Structural Biology, Institute of Biological Sciences, Federal University of Pará, Belém, Brazil; ^2^Department of Pediatric Dentistry and Orthodontics, School of Dentistry, Federal University of Rio de Janeiro, Rio de Janeiro, Brazil; ^3^Faculty of Medicine and Dentistry, School of Dentistry, University of Alberta, Edmonton, AB, Canada

**Keywords:** masticatory dysfunction, tooth loss, cognitive deficit, meta-analysis, systematic review

## Abstract

**Background:** An amount of cognition decline is normal with aging; however, intrinsic and extrinsic risk factors may exacerbate it, affecting social and occupational tasks. Masticatory dysfunction (MD), as a general term, refers to an impairment in the masticatory function triggered by a structural factor, such as tooth loss; functional factors, such as weaker bite force or a poorer masticatory performance; or both factors. MD acting as a source of chronic stress, promotes functional and morphological changes on the hippocampus, a brain area crucial for learning and memory abilities. This study aimed to synthesize evidence on the association between MD and cognitive deficit (CD), and demonstrate whether might be adequately considered as a risk factor.

**Methods:** Observational studies were screened in seven online databases; the search strategy (PECO) was focused in observational studies with humans as a population (P), presenting groups exposed (E), and non-exposed (C) to tooth loss, in which cognition parameters were measured and compared between groups (O). The final selection included only those studies comparing the effect in cognition between subjects having ≥20 remaining teeth and <20 remaining teeth, considering the latter as a structural factor triggering MD by the literature. Searching and data extraction were conducted following PRISMA guidelines. Qualitative and risk of bias evaluations were performed. The meta-analysis (MA) was constructed including the odds ratio (OR) and its 95% confidence interval (CI) comparing two groups—with/without MD. The level of evidence was rated by Grading Recommendations Assessment, Development and Evaluation (GRADE) approach.

**Results:** In total, 5,666 citations were identified, 14 accomplished our eligibility criteria, and nine were include in the MA. The MA demonstrates that individuals with MD had 46% higher chance to presented CD (OR 2.24 [1.73, 2.90], *p* < 0.00001, *I*^2^ = 46%). The level of evidence was rated as low by GRADE.

**Conclusion:** Despite the low certainty in evidence, according to our MA, MD is positively associated with increased risk of CD. However, more studies including other factors underlying MD and similar measurements should be conducted to obtain a strong estimate of the risk.

## Introduction

An optimal cognition status is essential to lead a quality of life (QoL) in young and old people. In 2010, 35.6 million people worldwide were estimated living with dementia and reach of 115.4 million people by 2050, is expected (Marchesi, 2011). Changes in cognition occur with normal aging (Harada et al., 2013), but they can be maximized by intrinsic and extrinsic risk factors (Marchesi, 2011), thus triggering severe cognition impairment that affect social and occupational tasks (Hugo and Ganguli, 2014). Cognitive changes are mostly related to declines of learning, memory, thinking, reasoning, and judgment abilities, such cases of dementia, among others neurocognitive disorders (Murman, 2015).

Influenceable external risk factors identified to the onset of neurocognitive disorders include genetic factors, hypertension, diabetes mellitus, hyperlipidemia, vascular disease, demographic, and lifestyle factors—such as smoking and alcohol use—and oxidative stress (Marchesi, 2011). On the other side, factors such as education, physical exercise, and active social engagement help to maintain cognition (Campbell et al., 2013). However, all the factors related to cognitive changes are still unknown and subject to study.

In this scenario, optimal mastication has been appeared as a factor to preserve cognitive functions, in addition to its well-known important role in food intake and swallowing (Teixeira et al., 2014). Masticatory dysfunction (MD), as a general term, refers to an impairment in the masticatory function triggered either by a structural factor, such as tooth loss; or by functional factors such as weaker bite force or a poorer masticatory performance (Ikebe et al., 2012; Lin, 2018), for example as in advance loss of periodontal support and tooth movement (Kosaka et al., 2014). Maximum bite force and masticatory ability scores are lower in older subjects with <20 remaining teeth (Tatematsu et al., 2004), suggesting a directly proportional association between tooth loss and inappropriate mastication (Ikebe et al., 2012).

MD such as a result from structural or functional factors, acting as a source of chronic stress, triggers functional and morphological changes on the hippocampus—a brain area crucial for learning and memory abilities (Azuma et al., 2017). These cognitive changes are promoted through the activation of the hypothalamic-pituitary-adrenal (HPA) axis and its glucocorticoid production (Azuma et al., 2017), similar to those changes induced by long term exposure to excessive corticosterone (Kubo et al., 2010).

In this regard, there are several studies around this association with different variables and outcome measures which become complicated the interpretation of the results in many systematic reviews made before (Tada and Miura, 2017). Thus, in order to reduce heterogeneity, the aim of this study was synthesizing only those studies that made a comparison between subjects with presence/absence of tooth loss as a structural factor triggering MD and its association with CD; and also demonstrate, through a quantitative analysis, whether MD might be adequately considered as a risk factor for CD, measuring its impact.

## Materials and Methods

### Protocol and Registration

The protocol for our systematic review was registered on the Prospective International Registry of Systematic Reviews—PROSPERO, under the registration number: CRD42016038269. The study was developed following the PRISMA (Preferred Reporting Items for Systematic Reviews and Meta-Analyses) guidelines (Moher et al., 2009). All items of the requirements are described in Supplementary Material 1.

### Eligibility Criteria and Search Strategy

Our search strategy, inclusion/exclusion criteria, and data extraction process were guided by the PECO question. Our PECO was focused in observational studies presenting humans as a population (P), presenting groups exposed (E), and non-exposed (C) to tooth loss, in which cognition parameters were measured and compared between groups (O). The final selection to evaluate the association of MD with the risk of CD, included only those studies comparing the effect in cognition between individuals having ≥20 remaining teeth (mild or no tooth loss) and <20 remaining teeth (severe tooth loss), considering the latter as a structural factor triggering MD. Thus, this review was developed based on the following guide question: “Is there any association between MD and CD?”

Although, there are also functional factors concerning chewing performance such as occlusal disharmony, bite force, soft-diet feeding, and salivary (Azuma et al., 2017), we established the number of remaining teeth as an inclusion criterion for presence/absence of tooth loss as a structural factor that promote MD, as a strategy to reduce heterogeneity in the measure of the chance of CD. Subjects having <20 remaining teeth reported inappropriate mastication compared with those having ≥20 remaining teeth with a better masticatory performance (Tatematsu et al., 2004; Savoca et al., 2010; Kosaka et al., 2014).

Following the inclusion criteria, observational studies with no publication date restrictions, nor age nor language neither, were included. To rejected studies, the exclusion criteria included systematic reviews and meta-analyses, comprehensive reviews articles, case reports, descriptive studies, opinion articles, technical articles, guidelines, animal, and *in vitro* studies.

In order to track all the citations existing about our question, five leading electronic databases used on health sciences research were accessed: PubMed, Scopus, Web of Science, Lilacs, and Cochrane. The OpenGrey and Google Scholar were also assessed as part of a gray literature search. The MeSH terms, keywords and search strategies were adapted according to the specificities of each database, covering terms related to tooth loss and cognitive decline. The selected terms were combined using Boolean operators (OR, AND). References of the studies were checked manually in order to seek other records that were not detected on the searching. The search strategy for each database is detailed in Supplementary Material 2.

Papers were tracked until February 2019. An alert of publication was set up on each database in order to be notified weekly for any articles from the new issues about our question. The citations found on the databases were entered into a reference management software (EndNote®, version X7, Thomson Reuters, Philadelphia, USA). Duplicates were identified—both automatic and manual—and then excluded.

The studies were selected independently by two authors (MA and RF), firstly filtering by title and abstract and lastly by the full text reading. A third author (RL) was consulted when there was disagreement about studies inclusion on this present review.

### Data Extraction

The data required of our final selection were extracted and tabulated based on the following items: year of publication, study design, characteristics of sample (size and origin), age, method of tooth loss measurement, diagnostic criteria for cognitive performance screening, statistical analysis and outcomes. Data extraction summary was tabulated on Table 1.

**Table 1:** Summary of characteristics of the included studies.

**Author, year/study design**	**Sample**			**Methods**		**Covariates**	**Statistical analysis**	**Outcomes**
	**Source**	**Size**	**Age**	**Cognitive measures**	**Tooth loss measures**			
Gao et al., 2016/Cs	China	*N* = 905 *n* = 651 (≥20 remaining teeth) *n* = 254: (<20 remaining teeth)	≥50	MMSE	A specially trained dentist from the Peking University School of Stomatology, China performed the dental examination	Age, sex, marriage status, family income, educational level, hyperlipidemia, hypertension, diabetes, stroke, and drinking/smoking/regular exercise habits	Multiple linear regression	Tooth loss and mitochondrial gene variants may have an effect on cognitive function in this study population
Gil-Montoya et al., 2015/Cs	Spain	*N* = 409 *n* = 153 (≥20 remaining teeth) *n* = 256 (<20 remaining teeth)	≥50	Phototest cognitive test	A complete clinical oral examination was performed by four dentists (SLI and three collaborator)	Age, sex, educational level, tobacco and alcohol consumption, hyperlipidemia, hyperglycemia, and family, personal, medical, clinical attachment loss and pharmacological histories related to cognitive impairment, i.e., potential confounders	Multiple logistic regression analysis	The significant association of cognitive impairment with the number of teeth present in the bivariate analysis disappeared after adjustment for age, sex, clinical attachment loss, oral hygiene habits, and the presence of hyperlipidemia Periodontitis appears to be associated with cognitive impairment after controlling for age, sex, educational level, and oral hygiene habits
Ishimiya et al., 2018/Cs	Japan	*N* = 334 *n* = 313 (≥20 remaining teeth) *n* = 21 (<20 remaining teeth)	≥60	DSM-III-R	Performed by a dentist in accordance with the methodology of the Third National Health and Nutrition Examination Survey	Age; sex; history of hypertension, diabetes mellitus, hyperlipidemia; low education	A one-way analysis of variance (ANOVA) and chi-square tests	Tooth loss-related dietary pattern was associated cognitive impairment in a general Japanese population
Nilsson et al., 2014/Cs	Sweden	*N* = 1090 *n* = 480 (≥20 remaining teeth) *n* = 610 (<20 remaining teeth)	≥60	MMSE and clock test	An oral examination including clinical registration of the number of teeth present was performed by a specially trained dental hygienist	Age and education	Simple logistic regression and multivariate logistic regression	A statistically significant association of the number of teeth on cognitive abilities of older adults were demonstrated when adjusted for age and level of education
Nilsson et al., 2018 Cs	Sweden	*N* = 1470 *n* = 767 (≥20 remaining teeth) *n* = 703 (<20 remaining teeth)	≥60	MMSE	Comprehensive clinical and radiographic examination by an experienced dental hygienist	Age, gender, and education	A chi-square test and multivariate logistic regression	Adjusted for age, gender, and level of education, a statistically significant association between loss of alveolar bone, the number of teeth and the outcome of the MMSE test was confirmed
Park et al., 2013/Cs	Korea	*N* = 438 *n* = 108 (≥20 remaining teeth) *n* = 330 (<20 remaining teeth)	≥50	MMSE	Study did not mention about the dental measure but showed a number of remaining teeth	Smoking, alcohol consumption, hypertension, diabetes, hyperlipidemia, stroke, dementia, and education levels	Independent *t*-tests and the chi-square test. The Pearson correlation and partial correlations analyses	The number of teeth lost is related to cognitive impairment
Reyes-Ortiz et al., 2013/PC	United States	*N* = 3032 *n* = 1122 (≥20 remaining teeth) *n* = 1910 (<20 remaining teeth)	≥65	MMSE	Self-reported	Age, Gender, Education level, Spanish spoken at interview, hypertension, stroke, diabetes, and heart attack	Mantel-Haenszel Chi-square test for categorical variables or the Kruskal-Wallis non-parametric ANOVA test for continuous variables	Fewer teeth predict cognitive decline in older Mexican Americans that are independent of socio-demographic variables and health factors such as visual impairment, medical conditions, and functional impairment
Saito et al., 2013/Cs	Japan	*N* = 462 *n* = 436 (≥20 remaining teeth) *n* = 26 (<20 remaining teeth)	≥60	MMSE	A dental examination was performed by two dentists under artificial lighting, with both the dentist and the subject in a seated position	The demographic (age, gender, education level) and lifestyle (smoking, drinking), positive history of diseases, TMIG-IC score, and CES-D	Student's unpaired *t*-test (for continuous variables) or the chi-square test (for categorical variables)	The number of teeth (0–10) was found to be a significant independent risk factor of cognitive impairment
Shimazaki et al., 2001/Cs	Japan	*N* = 122 *n* = 59 (≥20 remaining teeth) *n* = 63 (<20 remaining teeth)	≥65	Mental health status from medical records of each institution	Was examined under sufficient artificial light, with dental mirrors and explorers, by two dentists trained in the use of epidemiological indices for oral health	Age, physical health, type of institution, and cerebrovascular disorder	Logistic regression analysis	The relationship between baseline dentition status and follow-up mental impairment was not significant
Stewart et al., 2015/PC	England	*N* = 697 *n* = 448 (≥20 remaining teeth) *n* = 249 (<20 remaining teeth)	≥70	DSM-III-R	Dental status	Age, education, stroke, myocardial infarction, diabetes mellitus, smoking status, blood pressure, body mass index, and cholesterol level	Logistic regression models and separate regression models	Lower tooth count was a significant association for one of the three examinations
Takeuchi et al., 2015/Cs	Japan	*N* = 200 *n* = 20 (≥20 remaining teeth) *n* = 180 (<20 remaining teeth)	≥60	MMSE	Dental status was clinically examined by one trained dentist	Demographic and life style characteristics	Linear regression models	Loss of posterior teeth occlusion was independently associated with cognitive decline in nursing home older residents in Japan
Takeuchi et al., 2017/PC	Japan	*N* = 1014 *n* = 893 (≥20 remaining teeth) *n* = 673 (<20 remaining teeth)	≥75	DSM-III-R	Calibrated dentists performed a clinical oral examination, following the method of the Third National Health and Nutrition Examination Survey	Demographic characteristics, current occupation, medical history and treatment, physical activity, smoking habits, alcohol intake, tooth brushing frequency, and regular dental visits	Logistic regression analysis	Tooth loss is a risk factor for development of all-cause dementia and AD in an elderly Japanese population
Yamamoto et al., 2012/PC	Japan	*N* = 2919 *n* = 1299 (≥20 remaining teeth) *n* = 1620 (<20 remaining teeth)	≥65	Standardized questionnaire (the name was not specified)	Assessment by experts	Age, household income, presence of illness, alcohol intake, exercise, and forgetfulness	Univariate and multivariate models	Few teeth were associated with dementia in older Japanese people even after adjusting for sociodemographics, health status, health behaviors, and forgetfulness as an early symptom of mild cognitive impairment
Zhu et al., 2015/Cs	China	*N* = 161 *n* = 79 (≥20 remaining teeth) *n* = 82 (<20 remaining teeth)	≥60	MoCA Chinese version	The number of missing teeth, excluding the third molars, was recorded by a single investigator within 7 days of admission	Age, sex, body-mass index, education level, income, medical history, inflammatory markers, and medical assessment	Independent *t*-test, chi-squared analysis, and non-parametric test as appropriate. Logistic regression was used to identify the independent variables related with cognitive impairment	Multiple tooth loss is independently associated with vascular cognitive impairment in subjects with acute ischemic stroke

*Cs, Cross-sectional study; PC, Prospective cohort study; MMSE, Mini-Mental State Examination; MoCa, Montreal Cognitive Assessment; DSM-III-R, Diagnostic and Statistical Manual of Mental Disorders, Third edition, Revised*.

The extraction of data was performed independently and in duplicate by two authors (MA and RF). Differences were discussed together with a third author (RL).

### Risk of Bias in Individual Studies

To evaluate the methodological quality and the risk of bias, the checklist developed by Fowkes and Fulton (1991) was applied. This checklist has domains related to the design of the study and sample; characteristics of the control group; quality of measurements and outcomes; and completeness and distorting influences.

After evaluating each criterion, a signal (++) was assigned to cases where problems in the study were found or (+) for the minor problems. These evaluations helped to determine if the methods were adequate to produce consistent and valid information, as well as whether the results provided the expected effects. Those items where the question was not applicable to the type of study was assigned the acronym NA (not applicable). In cases with no problems found, the signal (0) was assigned (see the criteria of assessment in Supplementary Material 3).

Afterwards, the studies were analyzed for “bias results,” “confounding influences,” and “chance occurrences.” In attempt to determine the value of the study, three summary questions were answered: “Are the results erroneously biased in certain direction?”; “Are there any serious confounding or other distorting influences?” And “Is it likely that the results occurred by chance?". “YES” and “NO” answers were assigned. If the answer is NO in all three questions the article is considered reliable, with low risk of bias.

### Quantitative Synthesis (Meta-Analysis)

The extracted data was analyzed using the RevMan software (Review Manager, version 5.3, The Cochrane Collaboration; Copenhagen, Denmark) to assess the relationship between MD and CD. Only studies rated with low risk of bias were included in quantitative analysis. If some of the information needed for the meta-analysis was absent from any of the selected studies, the authors were contacted to provide the missing data.

In order to minimize the impact of confounding factors, a meta-analysis was constructed including the OR and its 95% CI comparing these two groups (Without MD/With MD) from included studies. This ratio measures effect as a log OR and the standard error of the log OR using generic inverse-variance weighting method. Combined results were presented as a pooled odds ratio.

Random-effect models were chosen because the studies were not functionally equivalent in which their objective to generalize the results from the meta-analysis and heterogeneity was tested using the *I*^2^ index. Sensitivity analyses were further conducted to estimate and verify the influence of studies, one by one, or grouped, on the pooled results if the heterogeneity was substantial or considerable (50–100%) (Higgins and Green, 2011). A funnel plot was generated to demonstrate possible publication bias.

### Level of Evidence

The quality of the evidence (certainty in the estimates of effect) was determined using the Grading of Recommendations Assessment, Development and Evaluation (GRADE) approach (Ryan and Hill, 2018). This tool offers a transparent and structured process for developing and presenting summaries of evidence, including its quality level, for systematic reviews (Guyatt et al., 2011).

In this assessment, observational studies start as low-quality evidence to support interventions. The quality of evidence is rating down, from low to very low quality, depending whether issues such as risk of bias, inconsistency, indirectness, imprecision, and publication bias, are serious or very serious. However, the quality of the evidence may be upgraded if the magnitude of effect is large or very large—considering the confounding factors—from very low to high (Guyatt et al., 2011).

## Results

### Characteristics of the Selected Studies

**Figure 1:**
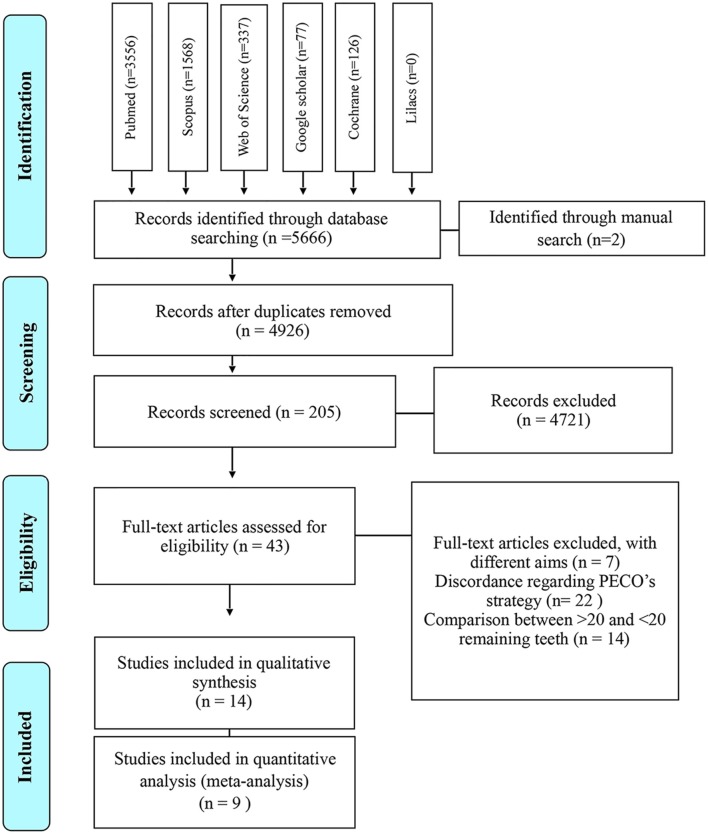
Flow diagram for study selection according with PRISMA statement.

The studies compiled and included in our qualitative and quantitative analysis was diagramed in Figure 1. As a result of our search strategy, 5,666 citations were identified. Two additional citations were identified through manual search. All these citations were entered into a reference management software (EndNote®, version X7, Thomson Reuters, Philadelphia, USA) and duplicates were identified and excluded, remaining 4,926 records.

Afterwards, citations were screened by title and abstract, 43 articles were selected for full-text reading and assessed for eligibility. Of these, seven were excluded for having different aims and 22 due to their discordance accordingly PECO's strategy (Supplementary Material 4). Finally, 14 observational studies−11 cross-sectional, two cohort and one prospective with longitudinal data, were included in qualitative synthesis (Shimazaki et al., 2001; Yamamoto et al., 2012; Park et al., 2013; Reyes-Ortiz et al., 2013; Saito et al., 2013; Nilsson et al., 2014, 2018; Gil-Montoya et al., 2015; Stewart et al., 2015; Takeuchi et al., 2015, 2017; Zhu et al., 2015; Gao et al., 2016; Ishimiya et al., 2018) but only nine—seven cross-sectional and two cohort (Shimazaki et al., 2001; Park et al., 2013; Saito et al., 2013; Gil-Montoya et al., 2015; Stewart et al., 2015; Zhu et al., 2015; Takeuchi et al., 2017; Ishimiya et al., 2018; Nilsson et al., 2018) accomplished all the characteristics needed and were included in the meta-analysis.

All of the records included had the comparison of the cognitive pattern between male and female, aged 50 years or above (older adults), with MD (<20 remaining teeth) and without MD (**≥**20 remaining teeth). Seven studies measured the cognitive pattern through the Mini-Mental State Examination (MMSE); three of them used the Diagnostic and Statistical Manual of Mental Disorders, Revised, Third Edition, Criteria (DSM-III R); the Montreal Cognitive Assessment (MoCA) was applied in one study; another one used the Phototest, a very recent method screened to evaluate cognition; two of the studies did not specify the validation of the cognitive assessment (Shimazaki et al., 2001; Yamamoto et al., 2012).

Although, most of the studies in our selection reported cognition disorders in individuals with <20 remaining teeth—with inappropriate mastication, two studies showed that this association disappeared after adjustment of covariates (Shimazaki et al., 2001; Gil-Montoya et al., 2015). One of them included clinical attachment lost as a confounding factor and demonstrates an association between periodontitis and cognition impairment after controlling for age, sex, educational level, and oral hygiene habits (Gil-Montoya et al., 2015). Another study shows an association between severe tooth loss and vascular cognitive impairment even after including inflammatory markers as a covariate in their statistical analysis (Zhu et al., 2015). Ishimiya et al. report that tooth-loss related dietary pattern is associated to cognitive impairment (Ishimiya et al., 2018). All the characteristics extracted from the studies were shown on Table 1.

### Qualitative Synthesis and Risk of Bias

In summary, all of the studies in our selection were rated having low risk of bias; however, some issues weakened the evidence. Nine studies do not describe the sampling method used, but it was not considered a serious problem due to their sample sizes are >50 (Yamamoto et al., 2012; Park et al., 2013; Saito et al., 2013; Stewart et al., 2015; Takeuchi et al., 2015, 2017; Zhu et al., 2015; Gao et al., 2016; Ishimiya et al., 2018).

Moreover, some studies had no description about randomization and did not make pairing of the groups neither, so then was rated as serious problem in this domain (Yamamoto et al., 2012; Reyes-Ortiz et al., 2013; Saito et al., 2013; Gil-Montoya et al., 2015; Stewart et al., 2015; Takeuchi et al., 2015, 2017; Gao et al., 2016; Ishimiya et al., 2018).

Most of the studies used evaluation methods validated and widely used to measure cognitive pattern, only three of them present problems in this domain; one used a questionnaire without validity details, but it was well-described to reproduce it (Yamamoto et al., 2012); another one used the data obtained in medical records without details of measurements methods (Shimazaki et al., 2001); a study used a relatively new screened method to evaluate cognitive pattern (Gil-Montoya et al., 2015). One study present quality control problems due to oral health status was based on self-reported data (Reyes-Ortiz et al., 2013).

In the domain about the distorting influences, all studies have problems with minor confounding factors not adjusted in their statistical analysis, but only in two of them were considered as a serious problem due to factors that might interfere in their results (Nilsson et al., 2014; Takeuchi et al., 2015). For that matter, Takeuchi et al. (2015), only included demographic and lifestyle characteristics, and Nilsson et al. (2018) adjusted only age and education, leaving out very important factors such as health conditions.

Based on the criteria, studies having an increased risk of bias were not considered. Although some issues considered as problems in the studies design—sampling method, group matching/randomization, validity and quality control—reduced the quality and strength of the evidence. All the studies of the final selection were rated as having low risk of bias as shown in Table 2.

**Table 2:** Assessment of quality and risk of bias for included studies.

**Guideline**	**Checklist**	**Gao et al., 2016**	**Gil-Montoya et al., 2015**	**Ishimiya et al., 2018**	**Nilsson et al., 2014**	**Nilsson et al., 2018**	**Park et al., 2013**	**Reyes-Ortiz et al., 2013**	**Saito et al., 2013**	**Shimazaki et al., 2001**	**Stewart et al., 2015**	**Takeuchi et al., 2015**	**Takeuchi et al., 2017**	**Yamamoto et al., 2012**	**Zhu et al., 2015**
Study design appropriate to objectives?	Objective common design														
	Prevalence cross-sectional														
	Prognosis cohort														
	Treatment controlled trial														
	Cause cohort, case-control, cross-sectional	0	0	0	0	0	0	0	0	0	0	0	0	0	0
Study sample representative?	Source of sample	0	0	0	0	0	0	0	0	0	0	0	0	0	0
	Sampling method	++	0	++	0	0	++	0	++	0	++	++	++	++	++
	Sample size	+	0	+	0	0	+	0	+	0	+	+	+	+	+
	Entry criteria/exclusion	0	0	0	0	0	0	0	0	0	0	0	0	0	0
	Non-respondents	0	0	0	0	0	0	0	0	0	0	0	0	0	0
Control group acceptable?	Definition of controls	0	0	0	0	0	0	0	0	0	0	0	0	0	0
	Source of controls	0	0	0	0	0	0	0	0	0	0	0	0	0	0
	Matching/randomization	++	+	+	0	0	0	+	++	0	+	++	+	+	0
	Comparable characteristics	0	0	0	0	0	0	0	0	0	0	0	0	0	0
Quality of measurements and outcomes?	Validity	0	+	0	0	0	0	++	0	+	0	0	0	+	0
	Reproducibility	0	0	0	0	0	0	0	0	+	0	0	0	+	0
	Blindness	0	0	0	0	0	0	0	0	0	0	0	0	0	0
	Quality control	0	0	0	0	0	0	0	0	0	0	0	0	0	0
Completeness	Compliance	0	0	0	0	0	0	0	0	0	0	0	0	0	0
	Drop outs	0	0	0	0	0	0	0	0	0	0	0	0	0	0
	Deaths	NA	NA	NA	NA	NA	NA	NA	NA	NA	NA	NA	NA	NA	NA
	Missing data	0	0	0	0	0	0	0	0	0	0	0	0	0	0
Distorting influences?	Extraneous treatments	0	0	0	0	0	0	0	0	0	0	0	0	0	0
	Contamination	NA	NA	NA	NA	NA	NA	NA	NA	NA	NA	NA	NA	NA	NA
	Changes over time	0	0	0	0	0	0	0	0	0	0	0	0	0	0
	Confounding factors	+	+	+	++	+	+	+	+	+	+	++	+	+	+
	Distortion reduced by analysis	0	0	0	0	0	0	0	0	0	0	0	0	0	0
Summary questions	Bias														
	Are the results erroneously biased in certain direction?	NO	NO	NO	NO	NO	NO	NO	NO	NO	NO	NO	NO	NO	NO
	Confounding														
	Are there any serious confusing or other distorting influences?	NO	NO	NO	YES	NO	NO	NO	NO	NO	NO	YES	NO	NO	NO
	Chance														
	Is it likely that the results occurred by chance?	NO	NO	NO	NO	NO	NO	NO	NO	NO	NO	NO	NO	NO	NO

*++, cases with problems; +, cases with minor problems; 0, cases with no problems; NA, not applicable*.

### Quantitative Analysis Through Meta-Analysis and Evidence Analysis—GRADE

Nine studies with low risk of bias were included in quantitative synthesis (Shimazaki et al., 2001; Park et al., 2013; Saito et al., 2013; Gil-Montoya et al., 2015; Stewart et al., 2015; Zhu et al., 2015; Takeuchi et al., 2017; Ishimiya et al., 2018; Nilsson et al., 2018).

**Figure 2:**
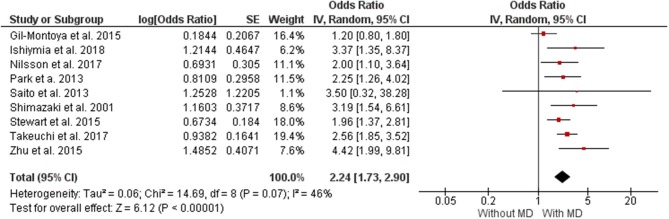
Forest plot of log OR between masticatory dysfunction and cognitive deficit.

Figure 2 presents data on the direction of the association between MD and CD. The analysis showed that individuals with MD had a 46% higher chance of presenting CD (OR 2.24 [1.73, 2.90], *p* < 0.00001, *I*^2^ = 46%) and non-significant (*I*^2^ *p* = 0.07) was significant *P* < 0.00001. The analysis of publication bias is represented in a funnel plot graphic on Supplementary Material 5.

The level of evidence was performed according to the data obtained in the meta-analysis. Nine studies were included in the meta-analysis. The results show general certainty of evidence LOW, with Relative effect (95% CI) of OR 2.24 (1.73–2.90). Table 3 shows all the results found in the GRADE evaluation.

**Table 3:** Summary of findings on the association between masticatory dysfunction and cognitive deficit.

**Certainty assessment**							**Effect**		**Certainty**
**No. of studies**	**Study design**	**Risk of bias**	**Inconsistency**	**Indirectness**	**Imprecision**	**Other considerations**	**Relative (95% CI)**	**Absolute (95% CI)**	
9 observational studies	Observational studies	Not serious	Not serious	Not serious	Serious^a^	Strong association	OR 2.24 (1.73–2.90)	–	⊕⊕○○ Low
								0 fewer per 1,000 (from 0 fewer to 0 fewer)	

*CI, Confidence interval; OR, Odds ratio*.

a *Upper limit of confidence interval is >25% of overall OR*.

## Discussion

This systematic review and meta-analysis aimed to synthetize only those studies comparing the cognition performance between subjects having ≥20 remaining teeth (mild or no tooth loss) and <20 remaining teeth (severe tooth loss), considering the latter as a structural factor that promote MD. In total, 14 studies with a low risk of bias were systematically selected, in which nine were included in the meta-analysis. The quantitative analysis of findings performed by our MA shows that individuals with inappropriate mastication have a greater chance of presenting a lower cognitive performance, suggesting that MD might be considered as a risk factor of CD.

Frequently, in order to synthesize the highest level of evidence existing in literature, rigorously systematic reviews, and meta-analysis are conducted in healthcare research. They are made following a well-designed methodological protocol, thus providing a precise estimate of the effect of risk factors for diseases. The estimate is obtained measuring the heterogeneity of the studies through statistical analyses. Therefore, this sort of reviews can provide the most trustworthy evidence for clinical decisions (Haidich, 2010).

In this context, we conducted this SR based on a search strategy previously designed and seven leading databases in medical research were accessed. Our literature search strategy and criteria for the inclusion/exclusion of studies were guided with the PECO question. According to our inclusion criterion, the final selection to evaluate the association of MD with the risk of CD, included only those studies in which was possible to extract the data concerning the comparison of the effect in cognition between subjects having ≥20 remaining teeth (mild or no tooth loss) and <20 remaining teeth (severe tooth loss) (Table 1), being the latter a structural factor that promote MD (Zhang et al., 2019).

MD, in general, refers to an impairment in the masticatory function due to structural factors, such as tooth loss, or functional factors such as weaker bite force or a poorer masticatory performance (Ikebe et al., 2012; Lin, 2018). Several studies have reported a decrease of masticatory function with increasing degree of tooth loss (Boretti et al., 1995; Savoca et al., 2010; Lexomboon et al., 2012). Tooth loss and also inappropriate vertical dimensions of crowns, bridges, or dentures can induce MD in humans (Miura et al., 1997). Studies that evaluated tooth loss and its impact in QoL have demonstrated that subjects having fewer than 20 teeth get more impact in QoL than those having 21–32 (Petersen et al., 2005; Tsakos et al., 2006; Akpata et al., 2011). Moreover, limited food choices due to dental function were significantly associated with having fewer remaining teeth (<20 remaining teeth) (Wang et al., 2014).

Although there are also functional factors regarding MD as occlusal disharmony, bite force, soft-diet feeding, and salivary (Azuma et al., 2017), literature supports that multiple tooth loss (<20 remaining teeth) by itself promotes an inappropriate chewing function (Tatematsu et al., 2004; Savoca et al., 2010; Kosaka et al., 2014). Maximum bite force and masticatory ability score in a study in patients having <20 remaining teeth, were lower; there was a strong positive correlation between the number of remaining teeth and the occlusal supporting score in this population (Tatematsu et al., 2004). In addition, other study in which occlusal force was significantly associated with masticatory performance, the number of residual teeth was significantly associated with masticatory performance in subjects having contact in four support zones and those having one to three support zones in the anterior region only (Ikebe et al., 2012).

However, it has also been suggested that it is important not only the number of remaining teeth but also the location of missing teeth for masticatory function assessment (Zhang et al., 2019). An unfavorable chewing ability is more prevalent in those individuals with <10 natural teeth in each jaw than those having all-natural teeth or more than 10 teeth in each jaw and the presence of three to four premolars can still provide good mastication function (Zhang et al., 2019). After variables controlled, the masticatory function is significantly associated with posterior occlusal contact (Ikebe et al., 2012).

Despite the studies included in this review did not report the location of missing teeth, several studies assessing the incidence of tooth loss have demonstrated that the posterior occluding units are the most affected and the front teeth the least affected (Montandon et al., 2012; Minja et al., 2016); being premolars less affected than molars (Silva-Junior et al., 2017), and this may interfere in the prediction of MD. Thus, future studies should include the location of missing teeth in dental records, considering that the presence of three to four premolars can still provide good mastication function, as well as the presence of removable full or partial dentures (Zhang et al., 2019), fixed dentures and implants (Prithviraj et al., 2014; Neves et al., 2015).

Although, there are also functional factors which can trigger MD, we decided to use only the data about the structural factor, multiple tooth loss (<20 remaining teeth)—in which literature supports that by itself promote MD (Tatematsu et al., 2004; Savoca et al., 2010; Kosaka et al., 2014)—as a strategy to reduce heterogeneity in the measure of the chance of CD, due to there are different variables and outcome measures among the studies evaluating tooth loss and/or MD and cognition.

Mastication is essential not only for food intake and swallowing, it has been reported that optimal chewing is also important preserving and promoting general health, including cognitive functions (Azuma et al., 2017). A study described the association between the loss of molars in senile rats and spatial memory deficits. Animals without molar teeth showed worse performance in behavioral tests than control animals maintained on a solid diet. Thus, the results of this experimental study suggested that the failure of spatial memory may be caused by inappropriate mastication and not only by tooth loss (Kato et al., 1997). MD associated with few residual molars, promote morphologic changes in the hippocampus and cerebral cortex, through increased blood flow in the bilateral lower frontal and parietal lobes during mastication activating various areas related with memory and learning ability (Kubo et al., 2010). MD acting as a source of chronic stress activates the HPA axis. The main neuroendocrine response to stress is via activation of the HPA axis, which stimulates—as a final product—the synthesis, and secretion of glucocorticoid (GC) from the adrenal cortex. Appropriate activation of the HPA axis is critically important for stress adaptation but repeated or prolonged HPA axis hyperactivity is linked with cognition disorders (Azuma et al., 2017). This process is similar to that induced by long term exposure to excessive corticosterone (Kubo et al., 2010).

The hypothesis of oxidative stress involvement and mitochondrial dysfunction in the pathogenesis of cognitive impairment and other diseases has been reported in several studies (Kann and Kovács, 2007). Oxidative phosphorylation occurring in the mitochondria is a major source of energy and this process produces free radicals or reactive oxygen species (Pero et al., 1990), in which, moderate or low levels are considered essential for neuronal development and function, whereas high levels are perilous (Wang et al., 2014). A community-based study in China, included in our review, found that the number of missing teeth and gene variations in the mitochondrial DNA D-loop region (including SNPs and haplogroups) were correlated with cognitive function in this population (Gao et al., 2016).

Nonetheless, one study reported no difference between subjects with many missing teeth compared to those with few missing teeth after adjusted cognition scores. The same study reported that subjects with periodontal inflammation (PI) obtained lower mean cognition scores compared to subjects without PI and the association of PI with cognitive scores was dependent on the number of missing teeth (Kamer et al., 2012). Furthermore, other study reports an effect of periodontal status and occlusal support on masticatory performance (Kosaka et al., 2014). In this regard, periodontitis has been also associated with cognitive functions due to the inflammatory and immune pathways involving in the pathogenesis of periodontitis (Noble et al., 2014; Nascimento et al., 2019). Tooth loss and gingival bleeding were markers of poorer executive function among dentate people (Naorungroj et al., 2013).

In this regard, a study included in our selection found an association between severe tooth loss and vascular cognitive impairment, even including inflammatory markers as a covariate (Zhu et al., 2015). Although there exists the possibility of periodontitis interfering in the risk of CD, considering that tooth loss is also a consequence of severe periodontitis, many studies evaluating the prevalence of tooth loss reported the tooth decay as a main cause of tooth loss (60–70%) being consistent among the studies compared with other reasons (Minja et al., 2016); periodontal disease has been reported as a minor cause (around 15%) (Caldas et al., 2000).

In contrast, a study classifying subjects according to number of teeth (Group 1 <20 teeth vs. Group 2 > 20 teeth), reported a positive association between fewer remaining teeth and lower cognitive status as represented by MMSE scores <25; but a score 25 indicated normal cognitive function, suggesting that the elderly in both groups had normal cognitive function (Wang et al., 2014). When confounding factors were controlled, lower MMSE scores were not associated with the subjects in Group 1 (<20 remaining teeth). It is unclear whether having total or partial dentures was controlled in its analysis, considering that subjects in this group had more partial dentures (Wang et al., 2014). Moreover, one study in elders living in rural Ecuador, reported that persons with <10 remaining teeth scored significantly worse in MoCA test when compared with those with ≥10 teeth (Del Brutto et al., 2014).

Cognition, as such, is about information processing performed by our brain and in this processing, the hippocampus plays an important role for cognitive functions, such as learning and memory abilities. When the hippocampus suffers some alterations, may trigger cognitive deficits, in which worst cases affect the ability to pay attention, process information and respond to information quickly, remember and recall information, think critically, plan, organize, solve problems and even inability to initiate speech (Trivedi, 2006). In order to detect cognitive impairments, some tests are widely used for measure cognition, such as MMSE and MoCA—30-point questionnaire mainly assessing memory abilities, executive capacity, attention, concentration, language functions, and orientation (Siqueira et al., 2018).

Despite the heterogeneity of the studies, the positive trends of association were consistent among all studies. Nine studies were included in our meta-analysis, and seven found an association between MD and CD, using the MMSE and MoCA. Well-known risk factors for cognitive deficits such as demographic and lifestyle characteristics, as well as some health conditions, were considered to avoid potential confounding factors in most of the studies included (Ishimiya et al., 2018). One study included dietary habits and using reduced rank regression analysis showed that tooth-loss related dietary pattern was associated with cognitive impairment. However, the diet of this study population cannot be generalized to other populations (Ishimiya et al., 2018).

Gil-Montoya et al. (2015), used Clinical Attachment Loss (CAL) in its correlation analysis—besides the other common variables—and the association between tooth loss and CD disappeared when all variables were adjusted. In addition, this study included CAL, because it also attempted to evaluate the association of periodontitis with CD, not tooth loss only, and as a result periodontitis actually appeared associated after controlling adjustments (Gil-Montoya et al., 2015).

Thus, our meta-analysis demonstrates that MD can be considered as a risk factor for CD. However, the level of evidence was rated as low by GRADE approach. First, due to the observational studies start as low-quality evidence to support interventions but also the amount of heterogeneity reported among the studies, mainly about the outcome measures of cognition. Therefore, according to GRADE parameters for grading, our evidence level regarding the estimated effect is not strong enough about direct effects of MD in cognition (Guyatt et al., 2011).

## Limitations

The main limitation in the estimate of the effect size is the heterogeneity of the studies related to the different outcome measures for CD and some confounding factors not included in some studies. Moreover, the absence of data among the studies about the location of missing teeth and other conditions related with reduction of mastication function such as occlusal disharmony, bite force, soft-diet feeding, and salivary flow, weakened the conclusions. The location of missing teeth may reduce the chance of risk of CD, due to the presence of two or three premolars can maintain mastication function.

## Conclusions

According to our findings, MD, predicted by a structural factor only, is associated with a chance of CD, and can be considered as a risk factor. However, the low level of evidence may complicate the precision of the results. Future studies including the location of remaining teeth, other factors related to masticatory performance and similar measures of cognition should be conducted to obtain a more precise estimate of the association of MD with CD risk.

We encourage researchers in health dentistry to use these findings to design and develop more studies in this association that can elucidate the chance of the CD risk and also more research about oral rehabilitation therapies that can restore the integrity of the masticatory system when it is lost. Moreover, it is important to spread the importance to preserve natural teeth to maintain general health, including cognition, in order to minimize or prevent neurocognitive disorders in older age.

## Data Availability

All datasets for this study are included in the manuscript and the Supplementary Files.

## Author Contributions

MA drafted the paper with input from all authors. RL, LM, and MA designed the study. MA and RF performed the searches, data extraction, quality assessment, analysis, and interpretation of data. MM, LM, and RF performed and interpreted the quantitative analysis. NF and RL revised the manuscript critically for important intellectual content and final approval of the version to be published.

### Conflict of Interest Statement

The authors declare that the research was conducted in the absence of any commercial or financial relationships that could be construed as a potential conflict of interest.

## References

[B1] Akpata E. Otoh E. Enwonwu C. Adeleke O. Joshipura K. (2011). Tooth loss, chewing habits, and food choices among older Nigerians in Plateau State: a preliminary study. Community Dent. Oral Epidemiol. 39, 409–415. 10.1111/j.1600-0528.2011.00612.x 21375560

[B2] Azuma K. Zhou Q. Niwa M. Kubo K.-Y. (2017). Association between Mastication, the Hippocampus, and the HPA Axis: a comprehensive review. Int. J. Mol. Sci. 18:E1687. 10.3390/ijms18081687 28771175 PMC5578077

[B3] Boretti G. Bickel M. Geering A. H. (1995). A review of masticatory ability and efficiency. J. Prosthet. Dent. 74, 400–403. 10.1016/S0022-3913(05)80381-6 8531159

[B4] Caldas A. Jr Marcenes W. Sheiham A. (2000). Reasons for tooth extraction in a Brazilian population. Int. Dent. J. 50, 267–273. 10.1111/j.1875-595X.2000.tb00564.x 15988885

[B5] Campbell N. L. Unverzagt F. Lamantia M. A. Khan B. A. Boustani M. A. (2013). Risk factors for the progression of mild cognitive impairment to dementia. Clin. Geriatr. Med. 29, 873–893. 10.1016/j.cger.2013.07.009 24094301 PMC5915285

[B6] Del Brutto O. H. Gardener H. Del Brutto V. J. Maestre G. E. Zambrano M. Montenegro J. E.. (2014). Edentulism associates with worse cognitive performance in community-dwelling elders in rural Ecuador: results of the Atahualpa project. J. Community Health 39, 1097–1100. 10.1007/s10900-014-9857-3 24627152 PMC11003771

[B7] Fowkes F. G. Fulton P. M. (1991). Critical appraisal of published research: introductory guidelines. BMJ. 302, 1136–1140. 10.1136/bmj.302.6785.1136 2043787 PMC1669795

[B8] Gao W. Wang X. Wang X. Cai Y. Luan Q. (2016). Association of cognitive function with tooth loss and mitochondrial variation in adult subjects: a community-based study in Beijing, China. Oral Dis. 22, 697–702. 10.1111/odi.12529 27353124

[B9] Gil-Montoya J. A. Sanchez-Lara I. Carnero-Pardo C. Fornieles F. Montes J. Vilchez R.. (2015). Is periodontitis a risk factor for cognitive impairment and dementia? A case-control study. J. Periodontol. 86, 244–253. 10.1902/jop.2014.140340 25345338

[B10] Guyatt G. Oxman A. D. Akl E. A. Kunz R. Vist G. Brozek J.. (2011). GRADE guidelines: 1. Introduction-GRADE evidence profiles and summary of findings tables. J. Clin. Epidemiol. 64, 383–394. 10.1016/j.jclinepi.2010.04.026 21195583

[B11] Haidich A. B. (2010). Meta-analysis in medical research. Hippokratia 14, 29–37. 21487488 PMC3049418

[B12] Harada C. N. Natelson Love M. C. Triebel K. L. (2013). Normal cognitive aging. Clin. Geriatr. Med. 29, 737–752. 10.1016/j.cger.2013.07.002 24094294 PMC4015335

[B13] Higgins J. Green S. (2011). Handbook for Systematic Reviews of Interventions, Version 5.1. 0 [updated March 2011]. The Cochrane Collaboration.

[B14] Hugo J. Ganguli M. (2014). Dementia and cognitive impairment: epidemiology, diagnosis, and treatment. Clin. Geriatr. Med. 30, 421–442. 10.1016/j.cger.2014.04.001 25037289 PMC4104432

[B15] Ikebe K. Matsuda K. I. Kagawa R. Enoki K. Okada T. Yoshida M.. (2012). Masticatory performance in older subjects with varying degrees of tooth loss. J. Dentistry 40, 71–76. 10.1016/j.jdent.2011.10.007 22037296

[B16] Ishimiya M. Nakamura H. Kobayashi Y. Noguchi-Shinohara M. Abe C. Dohmoto C.. (2018). Tooth loss-related dietary patterns and cognitive impairment in an elderly Japanese population: The Nakajima study. PLoS ONE 13:e0194504. 10.1371/journal.pone.0194504 29543872 PMC5854423

[B17] Kamer A. R. Morse D. E. Holm-Pedersen P. Mortensen E. L. Avlund K. (2012). Periodontal inflammation in relation to cognitive function in an older adult Danish population. J. Alzheimer's Dis. 28, 613–624. 10.3233/JAD-2011-102004 22045483

[B18] Kann O. Kovács R. (2007). Mitochondria and neuronal activity. Am. J. Physiol. Cell Physiol. 292, C641–657. 10.1152/ajpcell.00222.2006 17092996

[B19] Kato T. Usami T. Noda Y. Hasegawa M. Ueda M. Nabeshima T. (1997). The effect of the loss of molar teeth on spatial memory and acetylcholine release from the parietal cortex in aged rats. Behav. Brain Res. 83, 239–242. 10.1016/S0166-4328(97)86078-0 9062693

[B20] Kosaka T. Ono T. Yoshimuta Y. Kida M. Kikui M. Nokubi T.. (2014). The effect of periodontal status and occlusal support on masticatory performance: the Suita study. J. Clin. Periodontol. 41, 497–503. 10.1111/jcpe.12241 24527750

[B21] Kubo K. Y. Ichihashi Y. Kurata C. Iinuma M. Mori D. Katayama T.. (2010). Masticatory function and cognitive function. Okajimas Folia Anat. Jpn. 87, 135–140. 10.2535/ofaj.87.135 21174943

[B22] Lexomboon D. Trulsson M. Wårdh I. Parker M. G. (2012). Chewing ability and tooth loss: association with cognitive impairment in an elderly population study. J. Am. Geriatr. Soc. 60, 1951–1956. 10.1111/j.1532-5415.2012.04154.x 23035667

[B23] Lin C. S. (2018). Revisiting the link between cognitive decline and masticatory dysfunction. BMC Geriatr. 18:5. 10.1186/s12877-017-0693-z 29304748 PMC5756393

[B24] Marchesi V. T. (2011). Alzheimer's dementia begins as a disease of small blood vessels, damaged by oxidative-induced inflammation and dysregulated amyloid metabolism: implications for early detection and therapy. FASEB J. 25, 5–13. 10.1096/fj.11-0102ufm 21205781

[B25] Minja I. K. Astrom A. N. Masalu J. R. (2016). Prevalence and distribution of oral health knowledge according to sociodemographic, behavioural and clinical characteristics in selected coastal districts of Tanzania. Tanzan. J. Health Res. 18. 10.4314/thrb.v18i3.1

[B26] Miura H. Araki Y. Umenai T. (1997). Chewing activity and activities of daily living in the elderly. J. Oral Rehabil. 24, 457–460. 10.1046/j.1365-2842.1997.00530.x 9219992

[B27] Moher D. Liberati A. Tetzlaff J. Altman D. G. PRISMA Group (2009). Preferred reporting items for systematic reviews and meta-analyses: the PRISMA statement. PLoS Med. 6:e1000097 10.1371/journal.pmed.1000097 19621072 PMC2707599

[B28] Montandon A. Zuza E. De Toledo B. E. (2012). Prevalence and reasons for tooth loss in a sample from a dental clinic in Brazil. Int. J. Dent. 2012:719750. 10.1155/2012/719750 22973312 PMC3437633

[B29] Murman D. L. (2015). The impact of age on cognition. Semin. Hear. 36, 111–121. 10.1055/s-0035-1555115 27516712 PMC4906299

[B30] Naorungroj S. Schoenbach V. J. Beck J. Mosley T. H. Gottesman R. F. Alonso A.. (2013). Cross-sectional associations of oral health measures with cognitive function in late middle–aged adults: a community-based study. J. Am. Dent. Assoc. 144, 1362–1371. 10.14219/jada.archive.2013.0072 24282266 PMC4955404

[B31] Nascimento P. C. Castro M. M. L. Magno M. B. Carvalho A. P. C. P. S. (2019). Association between periodontitis and cognitive impairment in adults: a systematic review. Front. Neurol. 10:323. 10.3389/fneur.2019.00323 31105630 PMC6492457

[B32] Neves F. D. Mendes F. A. Borges T. D. F. Mendonça D. B. S. Prado M. M. D. S. Zancopé K. (2015). Masticatory performance with different types of rehabilitation of the edentulous mandible. Braz. J. Oral Sci. 14, 186–189. 10.1590/1677-3225v14n3a02

[B33] Nilsson H. Berglund J. Renvert S. (2014). Tooth loss and cognitive functions among older adults. Acta Odontol. Scand. 72, 639–644. 10.3109/00016357.2014.882983 24479559

[B34] Nilsson H. Berglund J. S. Renvert S. (2018). Periodontitis, tooth loss and cognitive functions among older adults. Clin. Oral Investig. 22, 2103–2109. 10.1007/s00784-017-2307-8 29270902

[B35] Noble J. M. Scarmeas N. Celenti R. S. Elkind M. S. Wright C. B. Schupf N.. (2014). Serum IgG antibody levels to periodontal microbiota are associated with incident Alzheimer disease. PLoS ONE 9:e114959. 10.1371/journal.pone.0114959 25522313 PMC4270775

[B36] Park H. Suk S. H. Cheong J. S. Lee H. S. Chang H. Do S. Y. (2013). Tooth loss may predict poor cognitive function in community-dwelling adults without dementia or stroke: the PRESENT project. J. Korean Med. Sci. 28, 1518–1521. 10.3346/jkms.2013.28.10.1518 24133359 PMC3792608

[B37] Pero R. W. Anderson M. W. Doyle G. A. Anna C. H. Romagna F. Markowitz M.. (1990). Oxidative stress induces DNA damage and inhibits the repair of DNA lesions induced by N-acetoxy-2-acetylaminofluorene in human peripheral mononuclear leukocytes. Cancer Res. 50, 4619–4625. 2114943

[B38] Petersen P. E. Bourgeois D. Ogawa H. Estupinan-Day S. Ndiaye C. (2005). The global burden of oral diseases and risks to oral health. Bull. World Health Organ. 83, 661–669. 16211157 PMC2626328

[B39] Prithviraj D. Madan V. Harshamayi P. Kumar C. G. Vashisht R. (2014). A comparison of masticatory efficiency in conventional dentures, implant retained or supported overdentures and implant supported fixed prostheses: a literature review. J. Dent. Implants 4:153–157. 10.4103/0974-6781.140882

[B40] Reyes-Ortiz C. A. Luque J. S. Eriksson C. K. Soto L. (2013). Self-reported tooth loss and cognitive function: data from the Hispanic Established Populations for Epidemiologic Studies of the Elderly (Hispanic EPESE). Colomb. Med. 44, 139–145. 24839334 PMC4002034

[B41] Ryan R. Hill S. (2018). How to GRADE the Quality of the Evidence. Cochrane Consumers and Communication Group, Version 3.0 (December 2016).

[B42] Saito Y. Sugawara N. Yasui-Furukori N. Takahashi I. Nakaji S. Kimura H. (2013). Cognitive function and number of teeth in a community-dwelling population in Japan. Ann. Gen. Psychiatry 12:20. 10.1186/1744-859X-12-20 23800274 PMC3706283

[B43] Savoca M. R. Arcury T. A. Leng X. Chen H. Bell R. A. Anderson A. M.. (2010). Severe tooth loss in older adults as a key indicator of compromised dietary quality. Public Health Nutr. 13, 466–474. 10.1017/S1368980009991236 19691903 PMC2847893

[B44] Shimazaki Y. Soh I. Saito T. Yamashita Y. Koga T. Miyazaki H.. (2001). Influence of dentition status on physical disability, mental impairment, and mortality in institutionalized elderly people. J. Dent. Res. 80, 340–345. 10.1177/00220345010800010801 11269726

[B45] Silva-Junior M. F. Batista M. J. De Sousa M. D. L. R. (2017). Incidence of tooth loss in adults: a 4-year population-based prospective cohort study. Int. J. Dent. 2017:6074703. 10.1155/2017/6074703 28785282 PMC5529659

[B46] Siqueira G. S. A. Hagemann P. M. S. Coelho D. S. Santos F. H. D. Bertolucci P. H. F. (2018). Can MoCA and MMSE be interchangeable cognitive screening tools? A systematic review. Gerontologist 20, 1–21. 10.1093/geront/gny126 30517634

[B47] Stewart R. Stenman U. Hakeberg M. Hägglin C. Gustafson D. Skoog I. (2015). Associations between oral health and risk of dementia in a 37-year follow-up study: the prospective population study of women in Gothenburg. J. Am. Geriatr. Soc. 63, 100–105. 10.1111/jgs.13194 25597561

[B48] Tada A. Miura H. (2017). Association between mastication and cognitive status: a systematic review. Arch. Gerontol. Geriatr. 70, 44–53. 10.1016/j.archger.2016.12.006 28042986

[B49] Takeuchi K. Izumi M. Furuta M. Takeshita T. Shibata Y. Kageyama S.. (2015). Posterior teeth occlusion associated with cognitive function in nursing home older residents: a cross-sectional observational study. PLoS ONE 10:e0141737. 10.1371/journal.pone.0141737 26512900 PMC4626072

[B50] Takeuchi K. Ohara T. Furuta M. Takeshita T. Shibata Y. Hata J.. (2017). Tooth loss and risk of dementia in the community: the Hisayama study. J. Am. Geriatr. Soc. 65, e95–e100. 10.1111/jgs.14791 28272750

[B51] Tatematsu M. Mori T. Kawaguchi T. Takeuchi K. Hattori M. Morita I.. (2004). Masticatory performance in 80-year-old individuals. Gerodontology 21, 112–119. 10.1111/j.1741-2358.2004.00018.x 15185992

[B52] Teixeira F. B. Fernandes Lde M. Noronha P. A. Dos Santos M. A. Gomes-Leal W. Maia Cdo S.. (2014). Masticatory deficiency as a risk factor for cognitive dysfunction. Int. J. Med. Sci. 11, 209–214. 10.7150/ijms.6801 24465167 PMC3894406

[B53] Trivedi J. (2006). Cognitive deficits in psychiatric disorders: current status. Indian J. Psychiatry 48, 10–20. 10.4103/0019-5545.31613 20703409 PMC2913637

[B54] Tsakos G. Steele J. G. Marcenes W. Walls A. W. Sheiham A. (2006). Clinical correlates of oral health-related quality of life: evidence from a national sample of British older people. Eur. J. Oral Sci. 114, 391–395. 10.1111/j.1600-0722.2006.00398.x 17026504

[B55] Wang T. F. Chen Y. Y. Liou Y. M. Chou C. (2014). Investigating tooth loss and associated factors among older Taiwanese adults. Arch. Gerontol. Geriatr. 58, 446–453. 10.1016/j.archger.2014.01.002 24568967

[B56] Yamamoto T. Kondo K. Hirai H. Nakade M. Aida J. Hirata Y. (2012). Association between self-reported dental health status and onset of dementia: a 4-year prospective cohort study of older Japanese adults from the Aichi Gerontological Evaluation Study (AGES) Project. Psychosom. Med. 74, 241–248. 10.1097/PSY.0b013e318246dffb 22408130

[B57] Zhang Q. Witter D. J. Bronkhorst E. M. Creugers N. H. J. (2019). The relationship between masticatory ability, age, and dental and prosthodontic status in an institutionalized elderly dentate population in Qingdao, China. Clin. Oral Investig. 23, 633–640. 10.1007/s00784-018-2477-z 29736683 PMC7736012

[B58] Zhu J. Li X. Zhu F. Chen L. Zhang C. Mcgrath C.. (2015). Multiple tooth loss is associated with vascular cognitive impairment in subjects with acute ischemic stroke. J. Periodont. Res. 50, 683–688. 10.1111/jre.12251 25495425

