# Supplementary material for: Masticatory Dysfunction by Extensive Tooth Loss as a Risk Factor for Cognitive Deficit: A Systematic Review and Meta-Analysis
Source: Front Physiol. 2019 Jul 3;10:832. doi: 10.3389/fphys.2019.00832 (PMC6618904; doi:10.3389/fphys.2019.00832)
Supplement: Supplementary file 1 [file Table_1.DOCX]

***Supplementary Material***

1. **PRISMA-P 2015 checklist**

| **Section/topic** | **#** | **Checklist item** | **Information reported** | | **Line number(s)** |
| --- | --- | --- | --- | --- | --- |
|  |  |  | **Yes** | **No** |  |
| **ADMINISTRATIVE INFORMATION** | | | | | |
| **Title** | | | | | |
| Identification | 1a | Identify the report as a protocol of a systematic review | x |  | 2 |
| Update | 1b | If the protocol is for an update of a previous systematic review, identify as such |  | x |  |
| **Registration** | 2 | If registered, provide the name of the registry (e.g., PROSPERO) and registration number in the Abstract | x |  | 91 |
| **Authors** | | | | | |
| Contact | 3a | Provide name, institutional affiliation, and e-mail address of all protocol authors; provide physical mailing address of corresponding author | x |  | 7-19 |
| Contributions | 3b | Describe contributions of protocol authors and identify the guarantor of the review | x |  | 447-450 |
| **Amendments** | 4 | If the protocol represents an amendment of a previously completed or published protocol, identify as such and list changes; otherwise, state plan for documenting important protocol amendments |  | x |  |
| **Support** | | | | | |
| Sources | 5a | Indicate sources of financial or other support for the review | x |  | 599 |
| Sponsor | 5b | Provide name for the review funder and/or sponsor | x |  | 444-445 |
| Role of sponsor/funder | 5c | Describe roles of funder(s), sponsor(s), and/or institution(s), if any, in developing the protocol | x |  | 442-450 |
| **INTRODUCTION** | | | | | |
| **Rationale** | 6 | Describe the rationale for the review in the context of what is already known | x |  | 51-79 |
| **Objectives** | 7 | Provide an explicit statement of the question(s) the review will address with reference to participants, interventions, comparators, and outcomes (PICO) | x |  | 80-87 |
| **METHODS** | | | | | |
| **Eligibility criteria** | 8 | Specify the study characteristics (e.g., PICO, study design, setting, time frame) and report characteristics (e.g., years considered, language, publication status) to be used as criteria for eligibility for the review | x |  | 96-106  123-125 |
| **Information sources** | 9 | Describe all intended information sources (e.g., electronic databases, contact with study authors, trial registers, or other grey literature sources) with planned dates of coverage | x |  | 126-133 |
| **Search strategy** | 10 | Present draft of search strategy to be used for at least one electronic database, including planned limits, such that it could be repeated | x |  | 133-138 |
| ***STUDY RECORDS*** | | | | | |
| Data management | 11a | Describe the mechanism(s) that will be used to manage records and data throughout the review | x |  | 143-146 |
| Selection process | 11b | State the process that will be used for selecting studies (e.g., two independent reviewers) through each phase of the review (i.e., screening, eligibility, and inclusion in meta-analysis) | x |  | 139-141  148-149 |
| Data collection process | 11c | Describe planned method of extracting data from reports (e.g., piloting forms, done independently, in duplicate), any processes for obtaining and confirming data from investigators | x |  | 140-141 |
| **Data items** | 12 | List and define all variables for which data will be sought (e.g., PICO items, funding sources), any pre-planned data assumptions and simplifications | x |  | 96-99 |
| **Outcomes and prioritization** | 13 | List and define all outcomes for which data will be sought, including prioritization of main and additional outcomes, with rationale | x |  | 197-231 |
| **Risk of bias in individual studies** | 14 | Describe anticipated methods for assessing risk of bias of individual studies, including whether this will be done at the outcome or study level, or both; state how this information will be used in data synthesis | x |  | 151-166 |
| ***DATA*** | | | | | |
| **Synthesis** | 15a | Describe criteria under which study data will be quantitatively synthesized | x |  | 168-183 |
|  | 15b | If data are appropriate for quantitative synthesis, describe planned summary measures, methods of handling data, and methods of combining data from studies, including any planned exploration of consistency (e.g., *I* ^2^, Kendall’s tau) | x |  | 173-177 |
|  | 15c | Describe any proposed additional analyses (e.g., sensitivity or subgroup analyses, meta-regression) | x |  | 180-183 |
|  | 15d | If quantitative synthesis is not appropriate, describe the type of summary planned |  | x |  |
| **Meta-bias(es)** | 16 | Specify any planned assessment of meta-bias(es) (e.g., publication bias across studies, selective reporting within studies) | x |  | 234-259 |
| **Confidence in cumulative evidence** | 17 | Describe how the strength of the body of evidence will be assessed (e.g., GRADE) | x |  | 185-194 |

1. **Search strategy for each database**

| **Databases** | **Combined** |
| --- | --- |
| PUBMED | ((((((((Humans[MeSH Terms]) OR Humans[Title/Abstract]) OR “Man (Taxonomy)”[Title/Abstract]) OR "Man, Modern”[Title/Abstract]) OR “Modern Man”[Title/Abstract]) OR “Human”[Title/Abstract]) OR “Homo sapiens"[Title/Abstract]) OR Adults[MeSH Terms]) OR Adult[Title/Abstract] AND (((((((((((((((((((((((((((((((((((((((((Tooth[MeSH Terms]) OR Tooth[Title/Abstract]) OR Teeth[Title/Abstract]) OR Dentition[MeSH Terms]) OR Dentition[Title/Abstract]) OR Dentitions[Title/Abstract]) OR “Tooth loss”[MeSH Terms]) OR “Tooth loss”[Title/Abstract]) OR “Loss, Tooth"[Title/Abstract]) OR “Mouth, Edentulous”[MeSH Terms]) OR “Mouth, Edentulous”[Title/Abstract]) OR “Edentulous Mouth”[Title/Abstract]) OR "Edentulous Mouths”[Title/Abstract]) OR “Mouth, Toothless”[Title/Abstract]) OR “Toothless Mouth"[Title/Abstract]) OR “Dentition, Permanent”[MeSH Terms]) OR “Dentition, Permanent”[Title/Abstract]) OR "Permanent Dentition”[Title/Abstract]) OR “Dentition, Secondary”[Title/Abstract]) OR “Secondary Dentition”[Title/Abstract]) OR “Dentition, Adult”[Title/Abstract]) OR “Adult Dentition”[Title/Abstract]) OR “Masticatory Muscles”[MeSH Terms]) OR "Masticatory Muscles”[Title/Abstract]) OR "Masticatory Muscle”[Title/Abstract]) OR “Muscle, Masticatory”[Title/Abstract]) OR "Muscles, Masticatory”[Title/Abstract]) OR “Bite Force”[MeSH Terms]) OR “Bite Forces”[Title/Abstract]) OR “Force, Bite”[Title/Abstract]) OR "Forces, Bite”[Title/Abstract]) OR “Occlusal Force”[Title/Abstract]) OR “Force, Occlusal”[Title/Abstract]) OR “Forces, Occlusal”[Title/Abstract]) OR “Occlusal Forces”[Title/Abstract]) OR “Masticatory Force”[Title/Abstract]) OR “Force, Masticatory”[Title/Abstract]) OR “Forces, Masticatory”[Title/Abstract]) OR “Masticatory Forces”[Title/Abstract]) OR Mastication[MeSH Terms]) OR Mastication[Title/Abstract]) OR Chewing[Title/Abstract] AND (((((((((((((((((((((((“Neurodegenerative Diseases”[MeSH Terms]) OR “Neurodegenerative Diseases”[Title/Abstract]) OR “Neurodegenerative Disease”[Title/Abstract]) OR “Degenerative Diseases, Neurologic”[Title/Abstract]) OR “Neurologic Degenerative Disease”[Title/Abstract]) OR "Degenerative Neurologic Diseases”[Title/Abstract]) OR “Degenerative Neurologic Disease”[Title/Abstract]) OR “Neurologic Disease, Degenerative”[Title/Abstract]) OR “Neurologic Diseases, Degenerative”[Title/Abstract]) OR "Nervous System Degenerative Diseases"[Title/Abstract]) OR “Neurodegenerative Disorders”[Title/Abstract]) OR “Neurodegenerative Disorder”[Title/Abstract]) OR “Neurologic Degenerative Conditions”[Title/Abstract]) OR “Degenerative Condition, Neurologic”[Title/Abstract]) OR “Degenerative Conditions, Neurologic”[Title/Abstract]) OR “Neurologic Degenerative Condition”[Title/Abstract]) OR “Neurologic Degenerative Diseases”[Title/Abstract]) OR “Degenerative Diseases, Nervous System”[Title/Abstract]) OR “Degenerative Neurologic Disorders”[Title/Abstract]) OR “Degenerative Neurologic Disorder”[Title/Abstract]) OR “Neurologic Disorder, Degenerative”[Title/Abstract]) OR “Neurologic Disorders, Degenerative”[Title/Abstract]) OR “Degenerative Diseases, Spinal Cord”[Title/Abstract]) OR “Degenerative Diseases, Central Nervous System”[Title/Abstract] OR (((((((((((((((((((((((((((((((((((((((((Dementia[MeSH Terms]) OR Dementias[Title/Abstract]) OR Amentia[Title/Abstract]) OR Amentias[Title/Abstract]) OR "Senile Paranoid Dementia”[Title/Abstract]) OR “Dementias, Senile Paranoid”[Title/Abstract]) OR “Paranoid Dementia, Senile”[Title/Abstract]) OR “Paranoid Dementias, Senile”[Title/Abstract]) OR “Senile Paranoid Dementias”[Title/Abstract]) OR "Familial Dementia”[Title/Abstract]) OR "Dementia, Familial”[Title/Abstract]) OR “Dementias, Familial”[Title/Abstract]) OR Amnesia[MeSH Terms]) OR Amnesia[Title/Abstract]) OR Amnesias[Title/Abstract]) OR “Amnesia-Memory Loss”[Title/Abstract]) OR “Amnesia Memory Loss”[Title/Abstract]) OR “Amnesia-Memory Losses”[Title/Abstract]) OR “Amnesia, Global”[Title/Abstract]) OR “Amnesias, Global”[Title/Abstract]) OR “Global Amnesia”[Title/Abstract]) OR “Global Amnesias”[Title/Abstract]) OR “Amnestic State”[Title/Abstract]) OR “Amnestic States”[Title/Abstract]) OR “State, Amnestic”[Title/Abstract]) OR “States, Amnestic”[Title/Abstract]) OR “Amnesia, Tactile”[Title/Abstract]) OR “Amnesias, Tactile”[Title/Abstract]) OR “Tactile Amnesia”[Title/Abstract]) OR “Tactile Amnesias”[Title/Abstract]) OR “Amnesia, Temporary”[Title/Abstract]) OR “Amnesias, Temporary”[Title/Abstract]) OR “Temporary Amnesia”[Title/Abstract]) OR "Temporary Amnesias”[Title/Abstract]) OR “Amnesia, Dissociative”[Title/Abstract]) OR “Amnesias, Dissociative”[Title/Abstract]) OR “Dissociative Amnesia”[Title/Abstract]) OR “Dissociative Amnesias”[Title/Abstract]) OR “Amnesia, Hysterical”[Title/Abstract]) OR “Amnesias, Hysterical”[Title/Abstract]) OR "Hysterical Amnesia”[Title/Abstract]) OR “Hysterical Amnesias”[Title/Abstract] OR (((((((((((((((((((((((((((((((((((((((((Cognition[MeSH Terms]) OR Cognition[Title/Abstract]) OR Cognitions[Title/Abstract]) OR “Cognitive Function”[Title/Abstract]) OR “Cognitive Functions”[Title/Abstract]) OR “Function, Cognitive”[Title/Abstract]) OR “Functions, Cognitive”[Title/Abstract]) OR “Cognition Disorders”[MeSH Terms]) OR “Cognition Disorders”[Title/Abstract]) OR “Disorder, Cognition”[Title/Abstract]) OR “Disorders, Cognition”[Title/Abstract]) OR Overinclusion[Title/Abstract]) OR “Cognitive Dysfunction[MeSH Terms]) OR “Cognitive Dysfunction”[Title/Abstract]) OR "Cognitive Dysfunctions”[Title/Abstract]) OR “Dysfunction, Cognitive”[Title/Abstract]) OR “Dysfunctions, Cognitive”[Title/Abstract]) OR “Cognitive Impairments”[Title/Abstract]) OR “Cognitive Impairment”[Title/Abstract]) OR “Impairment, Cognitive”[Title/Abstract]) OR “Impairments, Cognitive”[Title/Abstract]) OR “Mild Cognitive Impairment”[Title/Abstract]) OR “Cognitive Impairment, Mild”[Title/Abstract]) OR "Cognitive Impairments, Mild”[Title/Abstract]) OR “Impairment, Mild Cognitive”[Title/Abstract]) OR “Impairments, Mild Cognitive”[Title/Abstract]) OR “Mild Cognitive Impairments"[Title/Abstract]) OR “Mild Neurocognitive Disorder”[Title/Abstract]) OR “Disorder, Mild Neurocognitive”[Title/Abstract]) OR “Disorders, Mild Neurocognitive”[Title/Abstract]) OR “Mild Neurocognitive Disorders”[Title/Abstract]) OR “Neurocognitive Disorder, Mild”[Title/Abstract]) OR “Neurocognitive Disorders, Mild”[Title/Abstract]) OR “Cognitive Decline”[Title/Abstract]) OR “Cognitive Declines”[Title/Abstract]) OR “Decline, Cognitive”[Title/Abstract]) OR “Declines, Cognitive”[Title/Abstract]) OR “Mental Deterioration”[Title/Abstract]) OR “Deterioration, Mental”[Title/Abstract]) OR "Deteriorations, Mental"[Title/Abstract]) OR “Mental Deteriorations”[Title/Abstract]) OR ((((((((((((((((((((((((((“Memory Disorders”[MeSH Terms]) OR “Memory Disorders”[Title/Abstract]) OR “Memory Disorder”[Title/Abstract]) OR “Retention Disorders, Cognitive”[Title/Abstract]) OR "Cognitive Retention Disorder”[Title/Abstract]) OR “Cognitive Retention Disorders”[Title/Abstract]) OR “Retention Disorder, Cognitive”[Title/Abstract]) OR “Memory Loss"[Title/Abstract]) OR “Memory Losses”[Title/Abstract]) OR “Memory Disorder, Semantic”[Title/Abstract]) OR “Memory Disorders, Semantic”[Title/Abstract]) OR “Semantic Memory Disorders”[Title/Abstract]) OR “Semantic Memory Disorder”[Title/Abstract]) OR “Memory Disorder, Spatial”[Title/Abstract]) OR “Memory Disorders, Spatial"[Title/Abstract]) OR “Spatial Memory Disorders”[Title/Abstract]) OR “Spatial Memory Disorder”[Title/Abstract]) OR “Age-Related Memory Disorders”[Title/Abstract]) OR “Age Related Memory Disorders”[Title/Abstract]) OR “Age-Related Memory Disorder”[Title/Abstract]) OR “Memory Disorder, Age-Related”[Title/Abstract]) OR “Memory Disorders, Age-Related”[Title/Abstract]) OR "Memory Disorders, Age Related”[Title/Abstract]) OR “Memory Deficits”[Title/Abstract]) OR “Deficit, Memory”[Title/Abstract]) OR “Deficits, Memory”[Title/Abstract]) OR “Memory Deficit”[Title/Abstract] |
| Scopus | ( ( TITLE-ABS-KEY ( humans )  OR  TITLE-ABS-KEY ( "Man (Taxonomy)" )  OR  TITLE-ABS-KEY ( "Man, Modern" )  OR  TITLE-ABS-KEY ( "Modern Man" )  OR  TITLE-ABS-KEY ( human )  OR  TITLE-ABS-KEY ( "Homo sapiens" )  OR  TITLE-ABS-KEY ( adults )  OR  TITLE-ABS-KEY ( adult ) ) )  AND  ( ( TITLE-ABS-KEY ( tooth )  OR  TITLE-ABS-KEY ( teeth )  OR  TITLE-ABS-KEY ( dentition )  OR  TITLE-ABS-KEY ( dentitions )  OR  TITLE-ABS-KEY ( "Tooth loss" )  OR  TITLE-ABS-KEY ( "Loss, Tooth" )  OR  TITLE-ABS-KEY ( "Mouth, Edentulous" )  OR  TITLE-ABS-KEY ( "Edentulous Mouth" )  OR  TITLE-ABS-KEY ( "Edentulous Mouths" )  OR  TITLE-ABS-KEY ( "Mouth, Toothless" )  OR  TITLE-ABS-KEY ( "Toothless Mouth" )  OR  TITLE-ABS-KEY ( "Dentition, Permanent" )  OR  TITLE-ABS-KEY ( "Permanent Dentition" )  OR  TITLE-ABS-KEY ( "Dentition, Secondary" )  OR  TITLE-ABS-KEY ( "Secondary Dentition" )  OR  TITLE-ABS-KEY ( "Dentition, Adult" ) )  OR  ( ( TITLE-ABS-KEY ( "Adult Dentition" )  OR  TITLE-ABS-KEY ( "Masticatory Muscles" )  OR  TITLE-ABS-KEY ( "Masticatory Muscle" )  OR  TITLE-ABS-KEY ( "Muscle, Masticatory" )  OR  TITLE-ABS-KEY ( "Muscles, Masticatory" )  OR  TITLE-ABS-KEY ( "Bite Force" )  OR  TITLE-ABS-KEY ( "Bite Forces" )  OR  TITLE-ABS-KEY ( "Force, Bite" )  OR  TITLE-ABS-KEY ( "Forces, Bite" )  OR  TITLE-ABS-KEY ( "Occlusal Force" )  OR  TITLE-ABS-KEY ( "Force, Occlusal" )  OR  TITLE-ABS-KEY ( "Forces, Occlusal" )  OR  TITLE-ABS-KEY ( "Occlusal Forces" )  OR  TITLE-ABS-KEY ( "Masticatory Force" )  OR  TITLE-ABS-KEY ( "Force, Masticatory" )  OR  TITLE-ABS-KEY ( "Forces, Masticatory" )  OR  TITLE-ABS-KEY ( "Masticatory Forces" )  OR  TITLE-ABS-KEY ( mastication )  OR  TITLE-ABS-KEY ( chewing ) ) ) )  AND  ( ( ( TITLE-ABS-KEY ( "Memory Disorders" )  OR  TITLE-ABS-KEY ( "Memory Disorder" )  OR  TITLE-ABS-KEY ( "Retention Disorders, Cognitive" )  OR  TITLE-ABS-KEY ( "Cognitive Retention Disorder" )  OR  TITLE-ABS-KEY ( "Cognitive Retention Disorders" )  OR  TITLE-ABS-KEY ( "Retention Disorder, Cognitive" )  OR  TITLE-ABS-KEY ( "Memory Loss" )  OR  TITLE-ABS-KEY ( "Memory Losses" )  OR  TITLE-ABS-KEY ( "Memory Disorder, Semantic" )  OR  TITLE-ABS-KEY ( "Memory Disorders, Semantic" )  OR  TITLE-ABS-KEY ( "Semantic Memory Disorders" )  OR  TITLE-ABS-KEY ( "Semantic Memory Disorder" )  OR  TITLE-ABS-KEY ( "Memory Disorder, Spatial" )  OR  TITLE-ABS-KEY ( "Memory Disorders, Spatial" )  OR  TITLE-ABS-KEY ( "Spatial Memory Disorders" )  OR  TITLE-ABS-KEY ( "Spatial Memory Disorder" )  OR  TITLE-ABS-KEY ( "Age-Related Memory Disorders" )  OR  TITLE-ABS-KEY ( "Age Related Memory Disorders" )  OR  TITLE-ABS-KEY ( "Age-Related Memory Disorder" )  OR  TITLE-ABS-KEY ( "Memory Disorder, Age-Related" )  OR  TITLE-ABS-KEY ( "Memory Disorders, Age-Related" ) ) )  OR  ( TITLE-ABS-KEY ( "Memory Deficits" )  OR  TITLE-ABS-KEY ( "Deficit, Memory" )  OR  TITLE-ABS-KEY ( "Deficits, Memory" )  OR  TITLE-ABS-KEY ( "Memory Deficit" )  OR  TITLE-ABS-KEY ( cognition )  OR  TITLE-ABS-KEY ( cognitions )  OR  TITLE-ABS-KEY ( "Cognitive Function" )  OR  TITLE-ABS-KEY ( "Cognitive Functions" )  OR  TITLE-ABS-KEY ( "Function, Cognitive" )  OR  TITLE-ABS-KEY ( "Functions, Cognitive" )  OR  TITLE-ABS-KEY ( "Cognition Disorders" )  OR  TITLE-ABS-KEY ( "Disorder, Cognition" )  OR  TITLE-ABS-KEY ( "Disorders, Cognition" )  OR  TITLE-ABS-KEY ( "Overinclusion" )  OR  TITLE-ABS-KEY ( "Cognitive Dysfunction" )  OR  TITLE-ABS-KEY ( "Cognitive Dysfunctions" )  OR  TITLE-ABS-KEY ( "Dysfunction, Cognitive" )  OR  TITLE-ABS-KEY ( "Dysfunctions, Cognitive" )  OR  TITLE-ABS-KEY ( "Cognitive Impairments" )  OR  TITLE-ABS-KEY ( "Cognitive Impairment" )  OR  TITLE-ABS-KEY ( "Impairment, Cognitive" )  OR  TITLE-ABS-KEY ( "Impairments, Cognitive" )  OR  TITLE-ABS-KEY ( "Mild Cognitive Impairment" ) )  OR  ( ( TITLE-ABS-KEY ( "Cognitive Impairment, Mild" )  OR  TITLE-ABS-KEY ( "Cognitive Impairments, Mild" )  OR  TITLE-ABS-KEY ( "Impairment, Mild Cognitive" )  OR  TITLE-ABS-KEY ( "Impairments, Mild Cognitive" )  OR  TITLE-ABS-KEY ( "Mild Cognitive Impairments" )  OR  TITLE-ABS-KEY ( "Mild Neurocognitive Disorder" )  OR  TITLE-ABS-KEY ( "Disorder, Mild Neurocognitive" )  OR  TITLE-ABS-KEY ( "Disorders, Mild Neurocognitive" )  OR  TITLE-ABS-KEY ( "Mild Neurocognitive Disorders" )  OR  TITLE-ABS-KEY ( "Neurocognitive Disorder, Mild" )  OR  TITLE-ABS-KEY ( "Neurocognitive Disorders, Mild" )  OR  TITLE-ABS-KEY ( "Cognitive Decline" )  OR  TITLE-ABS-KEY ( "Cognitive Declines" )  OR  TITLE-ABS-KEY ( "Decline, Cognitive" )  OR  TITLE-ABS-KEY ( "Declines, Cognitive" )  OR  TITLE-ABS-KEY ( "Mental Deterioration" )  OR  TITLE-ABS-KEY ( "Deterioration, Mental" )  OR  TITLE-ABS-KEY ( "Deteriorations, Mental" )  OR  TITLE-ABS-KEY ( "Mental Deteriorations" )  OR  TITLE-ABS-KEY ( dementia )  OR  TITLE-ABS-KEY ( amentia )  OR  TITLE-ABS-KEY ( amentias ) ) )  OR  ( ( TITLE-ABS-KEY ( "Senile Paranoid Dementia" )  OR  TITLE-ABS-KEY ( "Dementias, Senile Paranoid" )  OR  TITLE-ABS-KEY ( "Paranoid Dementia, Senile" )  OR  TITLE-ABS-KEY ( "Paranoid Dementias, Senile" )  OR  TITLE-ABS-KEY ( "Senile Paranoid Dementias" )  OR  TITLE-ABS-KEY ( "Familial Dementia" )  OR  TITLE-ABS-KEY ( "Dementia, Familial" )  OR  TITLE-ABS-KEY ( "Dementias, Familial" )  OR  TITLE-ABS-KEY ( amnesia )  OR  TITLE-ABS-KEY ( amnesias )  OR  TITLE-ABS-KEY ( "Amnesia-Memory Loss" )  OR  TITLE-ABS-KEY ( "Amnesia Memory Loss" )  OR  TITLE-ABS-KEY ( "Amnesia-Memory Losses" )  OR  TITLE-ABS-KEY ( "Amnesia, Global" )  OR  TITLE-ABS-KEY ( "Amnesias, Global" )  OR  TITLE-ABS-KEY ( "Global Amnesia" )  OR  TITLE-ABS-KEY ( "Global Amnesias" )  OR  TITLE-ABS-KEY ( "Amnestic State" )  OR  TITLE-ABS-KEY ( "Amnestic States" )  OR  TITLE-ABS-KEY ( "State, Amnestic" )  OR  TITLE-ABS-KEY ( "States, Amnestic" )  OR  TITLE-ABS-KEY ( "Amnesia, Tactile" )  OR  TITLE-ABS-KEY ( "Amnesias, Tactile" )  OR  TITLE-ABS-KEY ( "Tactile Amnesia" )  OR  TITLE-ABS-KEY ( "Tactile Amnesias" ) ) )  OR  ( ( TITLE-ABS-KEY ( "Amnesia, Temporary" )  OR  TITLE-ABS-KEY ( "Amnesias, Temporary" )  OR  TITLE-ABS-KEY ( "Temporary Amnesia" )  OR  TITLE-ABS-KEY ( "Temporary Amnesias" )  OR  TITLE-ABS-KEY ( "Amnesia, Dissociative" )  OR  TITLE-ABS-KEY ( "Amnesias, Dissociative" )  OR  TITLE-ABS-KEY ( "Dissociative Amnesia" )  OR  TITLE-ABS-KEY ( "Dissociative Amnesias" )  OR  TITLE-ABS-KEY ( "Amnesia, Hysterical" )  OR  TITLE-ABS-KEY ( "Amnesias, Hysterical" )  OR  TITLE-ABS-KEY ( "Hysterical Amnesia" )  OR  TITLE-ABS-KEY ( "Hysterical Amnesias" )  OR  TITLE-ABS-KEY ( "Neurodegenerative Diseases" )  OR  TITLE-ABS-KEY ( "Neurodegenerative Disease" )  OR  TITLE-ABS-KEY ( "Degenerative Diseases, Neurologic" )  OR  TITLE-ABS-KEY ( "Neurologic Degenerative Disease" )  OR  TITLE-ABS-KEY ( "Degenerative Neurologic Diseases" )  OR  TITLE-ABS-KEY ( "Degenerative Neurologic Disease" )  OR  TITLE-ABS-KEY ( "Neurologic Disease, Degenerative" )  OR  TITLE-ABS-KEY ( "Neurologic Diseases, Degenerative" )  OR  TITLE-ABS-KEY ( "Nervous System Degenerative Diseases" ) ) )  OR  ( ( TITLE-ABS-KEY ( "Neurodegenerative Disorders" )  OR  TITLE-ABS-KEY ( "Neurodegenerative Disorder" )  OR  TITLE-ABS-KEY ( "Neurologic Degenerative Conditions" )  OR  TITLE-ABS-KEY ( "Degenerative Condition, Neurologic" )  OR  TITLE-ABS-KEY ( "Degenerative Conditions, Neurologic" )  OR  TITLE-ABS-KEY ( "Neurologic Degenerative Condition" )  OR  TITLE-ABS-KEY ( "Neurologic Degenerative Diseases" )  OR  TITLE-ABS-KEY ( "Degenerative Diseases, Nervous System" )  OR  TITLE-ABS-KEY ( "Degenerative Neurologic Disorders" )  OR  TITLE-ABS-KEY ( "Degenerative Neurologic Disorder" )  OR  TITLE-ABS-KEY ( "Neurologic Disorder, Degenerative" )  OR  TITLE-ABS-KEY ( "Neurologic Disorders, Degenerative" )  OR  TITLE-ABS-KEY ( "Degenerative Diseases, Spinal Cord" )  OR  TITLE-ABS-KEY ( "Degenerative Diseases, Central Nervous System" ) ) ) ) |
| LILACS | (tw:((tw:(Amnesia)) OR (tw:(Amnesia )) OR (tw:(Amnesias)) OR (tw:(“Amnesia-Memory Loss”)) OR (tw:(“Amnesia Memory Loss”)) OR (tw:(“Amnesia-Memory Losses”)) OR (tw:(“Amnesia, Global”)) OR (tw:( “Amnesias, Global”)) OR (tw:(“Global Amnesia”)) OR (tw:( “Global Amnesias”)) OR (tw:(“Amnestic State”)) OR (tw:(“Amnestic States”)) OR (tw:(“State, Amnestic”)) OR (tw:(“States, Amnestic”)) OR (tw:( “Amnesia, Tactile”)) OR (tw:( “Amnesias, Tactile”)) OR (tw:(“Tactile Amnesia” )) OR (tw:(“Tactile Amnesias”)) OR (tw:(“Amnesia, Temporary”)) OR (tw:(“Amnesias, Temporary”)) OR (tw:( “Temporary Amnesia”)) OR (tw:("Temporary Amnesias”)) OR (tw:(“Amnesia, Dissociative”)) OR (tw:(“Amnesias, Dissociative”)) OR (tw:( “Dissociative Amnesia”)) OR (tw:( “Dissociative Amnesias”)) OR (tw:(“Amnesia, Hysterical”)) OR (tw:(“Amnesias, Hysterical”)) OR (tw:( "Hysterical Amnesia”)) OR (tw:(“Hysterical Amnesias”)) OR (tw:(“Neurodegenerative Diseases”)) OR (tw:(“Neurodegenerative Diseases” )) OR (tw:(“Neurodegenerative Disease”)) OR (tw:(“Degenerative Diseases, Neurologic” )) OR (tw:(“Neurologic Degenerative Disease”)) OR (tw:("Degenerative Neurologic Diseases”)) OR (tw:( “Degenerative Neurologic Disease”)) OR (tw:( “Neurologic Disease, Degenerative”)) OR (tw:(“Neurologic Diseases, Degenerative”)) OR (tw:( "Nervous System Degenerative Diseases")) OR (tw:( “Neurodegenerative Disorders” )) OR (tw:( “Neurodegenerative Disorder”)) OR (tw:(“Neurologic Degenerative Conditions”)) OR (tw:(“Degenerative Condition, Neurologic”)) OR (tw:( “Degenerative Conditions, Neurologic”)) OR (tw:(“Neurologic Degenerative Condition”)) OR (tw:(“Neurologic Degenerative Diseases” )) OR (tw:(“Degenerative Diseases, Nervous System”)) OR (tw:( “Degenerative Neurologic Disorders” )) OR (tw:(“Degenerative Neurologic Disorder”)) OR (tw:(“Neurologic Disorder, Degenerative”)) OR (tw:(“Neurologic Disorders, Degenerative”)) OR (tw:(“Degenerative Diseases, Spinal Cord”)) OR (tw:(“Degenerative Diseases, Central Nervous System")) OR (tw:(“Cognition Disorders”)) OR (tw:(“Cognition Disorders” )) OR (tw:(“Disorder, Cognition”)) OR (tw:(“Disorders, Cognition”)) OR (tw:(Overinclusion)) OR (tw:(“Cognitive Dysfunction”)) OR (tw:(“Cognitive Dysfunction” )) OR (tw:("Cognitive Dysfunctions”)) OR (tw:(“Dysfunction, Cognitive”)) OR (tw:( “Dysfunctions, Cognitive”)) OR (tw:(“Cognitive Impairments”)) OR (tw:(“Cognitive Impairment”)) OR (tw:( “Impairment, Cognitive”)) OR (tw:( “Impairments, Cognitive” )) OR (tw:( “Mild Cognitive Impairment”)) OR (tw:( “Cognitive Impairment, Mild”)) OR (tw:( "Cognitive Impairments, Mild”)) OR (tw:(“Impairment, Mild Cognitive”)) OR (tw:( “Impairments, Mild Cognitive”)) OR (tw:(“Mild Cognitive Impairments" )) OR (tw:(“Mild Neurocognitive Disorder”)) OR (tw:( “Disorder, Mild Neurocognitive”)) OR (tw:( “Disorders, Mild Neurocognitive”)) OR (tw:( “Mild Neurocognitive Disorders”)) OR (tw:( “Neurocognitive Disorder, Mild”)) OR (tw:(“Neurocognitive Disorders, Mild”)) OR (tw:( “Cognitive Decline”)) OR (tw:( “Cognitive Declines”)) OR (tw:( “Decline, Cognitive”)) OR (tw:( “Declines, Cognitive”)) OR (tw:( “Mental Deterioration”)) OR (tw:( “Deterioration, Mental”)) OR (tw:("Deteriorations, Mental" )) OR (tw:( “Mental Deteriorations”)) OR (tw:(Dementia)) OR (tw:(Dementias )) OR (tw:( Amentia)) OR (tw:(Amentias)) OR (tw:("Senile Paranoid Dementia”)) OR (tw:( “Dementias, Senile Paranoid”)) OR (tw:(“Paranoid Dementia, Senile”)) OR (tw:(“Paranoid Dementias, Senile”)) OR (tw:(“Senile Paranoid Dementias”)) OR (tw:("Familial Dementia”)) OR (tw:( "Dementia, Familial”)) OR (tw:( “Dementias, Familial”)) OR (tw:(“Memory Disorders”)) OR (tw:(“Memory Disorders”)) OR (tw:(“Memory Disorder”)) OR (tw:(“Retention Disorders, Cognitive”)) OR (tw:("Cognitive Retention Disorder”)) OR (tw:(“Cognitive Retention Disorders” )) OR (tw:(“Retention Disorder, Cognitive”)) OR (tw:(“Memory Loss")) OR (tw:(“Memory Losses” )) OR (tw:(“Memory Disorder, Semantic”)) OR (tw:(“Memory Disorders, Semantic”)) OR (tw:( “Semantic Memory Disorders”)) OR (tw:(“Semantic Memory Disorder”)) OR (tw:(“Memory Disorder, Spatial” )) OR (tw:(“Memory Disorders, Spatial")) OR (tw:(“Spatial Memory Disorders”)) OR (tw:(“Spatial Memory Disorder”)) OR (tw:(“Age-Related Memory Disorders”)) OR (tw:(“Age Related Memory Disorders”)) OR (tw:(“Age-Related Memory Disorder” )) OR (tw:(“Memory Disorder, Age-Related”)) OR (tw:(“Memory Disorders, Age-Related”)) OR (tw:("Memory Disorders, Age Related”)) OR (tw:( “Memory Deficits”)) OR (tw:( “Deficit, Memory”)) OR (tw:(“Deficits, Memory” )) OR (tw:(“Memory Deficit”)) OR (tw:(Cognition)) OR (tw:(Cognition )) OR (tw:(Cognitions)) OR (tw:(“Cognitive Function”)) OR (tw:(“Cognitive Functions”)) OR (tw:( “Function, Cognitive”)) OR (tw:(“Functions, Cognitive”)) OR (tw:(“Cognition Disorders”)) OR (tw:(“Cognition Disorders” )) OR (tw:(“Disorder, Cognition”)) OR (tw:(“Disorders, Cognition”)) OR (tw:(Overinclusion)) OR (tw:(“Cognitive Dysfunction”)) OR (tw:(“Cognitive Dysfunction” )) OR (tw:("Cognitive Dysfunctions”)) OR (tw:(“Dysfunction, Cognitive”)) OR (tw:( “Dysfunctions, Cognitive”)) OR (tw:(“Cognitive Impairments”)) OR (tw:(“Cognitive Impairment”)) OR (tw:( “Impairment, Cognitive”)) OR (tw:( “Impairments, Cognitive” )) OR (tw:( “Mild Cognitive Impairment”)) OR (tw:( “Cognitive Impairment, Mild”)) OR (tw:( "Cognitive Impairments, Mild”)) OR (tw:(“Impairment, Mild Cognitive”)) OR (tw:( “Impairments, Mild Cognitive”)) OR (tw:(“Mild Cognitive Impairments" )) OR (tw:(“Mild Neurocognitive Disorder”)) OR (tw:( “Disorder, Mild Neurocognitive”)) OR (tw:( “Disorders, Mild Neurocognitive”)) OR (tw:( “Mild Neurocognitive Disorders”)) OR (tw:( “Neurocognitive Disorder, Mild”)) OR (tw:(“Neurocognitive Disorders, Mild”)) OR (tw:( “Cognitive Decline”)) OR (tw:( “Cognitive Declines”)) OR (tw:( “Decline, Cognitive”)) OR (tw:( “Declines, Cognitive”)) OR (tw:( “Mental Deterioration”)) OR (tw:( “Deterioration, Mental”)) OR (tw:("Deteriorations, Mental" )) OR (tw:( “Mental Deteriorations”)) OR (tw:(Dementia)) OR (tw:(Dementias )) OR (tw:( Amentia)) OR (tw:(Amentias)) OR (tw:("Senile Paranoid Dementia”)) OR (tw:( “Dementias, Senile Paranoid”)) OR (tw:(“Paranoid Dementia, Senile”)) OR (tw:(“Paranoid Dementias, Senile”)) OR (tw:(“Senile Paranoid Dementias”)) OR (tw:("Familial Dementia”)) OR (tw:( "Dementia, Familial”)) OR (tw:( “Dementias, Familial”)) AND (tw:(humans)) OR (tw:(humans)) OR (tw:("Man (Taxonomy)")) OR (tw:("Man, Modern")) OR (tw:("Modern Man")) OR (tw:(human)) OR (tw:("Homo sapiens")) OR (tw:(adults)) OR (tw:(adult)) AND (tw:(Tooth)) OR (tw:(Tooth )) OR (tw:(Teeth)) OR (tw:(Dentition)) OR (tw:(Dentition )) OR (tw:(Dentitions)) OR (tw:(“Tooth loss”)) OR (tw:("Tooth loss")) OR (tw:( “Loss, Tooth”)) OR (tw:(“Mouth, Edentulous”)) OR (tw:(“Mouth, Edentulous”)) OR (tw:(“Edentulous Mouth”)) OR (tw:("Edentulous Mouths”)) OR (tw:(“Mouth, Toothless”)) OR (tw:(“Toothless Mouth”)) OR (tw:(“Dentition, Permanent”)) OR (tw:(“Dentition, Permanent” )) OR (tw:( "Permanent Dentition”)) OR (tw:(“Dentition, Secondary”)) OR (tw:(“Secondary Dentition”)) OR (tw:( “Dentition, Adult”)) OR (tw:(“Adult Dentition”)) OR (tw:(“Masticatory Muscles”)) OR (tw:("Masticatory Muscles” )) OR (tw:("Masticatory Muscle”)) OR (tw:(“Muscle, Masticatory”)) OR (tw:("Muscles, Masticatory”)) OR (tw:(“Bite Force”)) OR (tw:(“Bite Forces” )) OR (tw:(“Force, Bite”)) OR (tw:("Forces, Bite”)) OR (tw:( “Occlusal Force” )) OR (tw:( “Force, Occlusal”)) OR (tw:( “Forces, Occlusal”)) OR (tw:(“Occlusal Forces”)) OR (tw:(“Masticatory Force”)) OR (tw:(“Force, Masticatory”)) OR (tw:(“Masticatory Forces”)) OR (tw:(Mastication)) OR (tw:(Mastication)) OR (tw:(Chewing)))) |
| COCHRANE | 'Humans or "Man (Taxonomy)" or "Man, Modern" or "Modern Man" or Human or "Homo sapiens" or Adults or Adult in Title, Abstract, Keywords and Tooth or Teeth or Dentition or Dentitions or "Tooth loss" or "Loss, Tooth" or "Mouth, Edentulous" or "Edentulous Mouth" or "Edentulous Mouths" or "Mouth, Toothless" or "Toothless Mouth" or "Dentition, Permanent" or "Permanent Dentition" or "Dentition, Secondary" or "Secondary Dentition" or "Dentition, Adult" or "Adult Dentition" or "Masticatory Muscles" or "Masticatory Muscle" or "Muscle, Masticatory" or "Muscles, Masticatory" or "Bite Force" or "Bite Forces" or "Force, Bite" or "Forces, Bite" or "Occlusal Force" or "Force, Occlusal" or "Forces, Occlusal" or "Occlusal Forces" or "Masticatory Force" or "Force, Masticatory" or "Forces, Masticatory" or "Masticatory Forces" or Mastication or Chewing in Title, Abstract, Keywords and "Memory Disorders" or "Memory Disorder" or "Retention Disorders, Cognitive" or "Cognitive Retention Disorder" or "Cognitive Retention Disorders" or "Retention Disorder, Cognitive" or "Memory Loss" or "Memory Losses" or "Memory Disorder, Semantic" or "Memory Disorders, Semantic" or "Semantic Memory Disorders" or "Semantic Memory Disorder" or "Memory Disorder, Spatial" or "Memory Disorders, Spatial" or "Spatial Memory Disorders" or "Spatial Memory Disorder" or "Age-Related Memory Disorders" or "Age Related Memory Disorders" or "Age-Related Memory Disorder" or "Memory Disorder, Age-Related" or "Memory Disorders, Age-Related" or "Memory Disorders, Age Related" or "Memory Deficits" or "Deficit, Memory" or "Deficits, Memory" or "Memory Deficit" or Cognition or Cognitions or "Cognitive Function" or "Cognitive Functions" or "Function, Cognitive" or "Functions, Cognitive" or "Cognition Disorders" or "Disorder, Cognition" or "Disorders, Cognition" or "Overinclusion" or "Cognitive Dysfunction" or "Cognitive Dysfunctions" or "Dysfunction, Cognitive" or "Dysfunctions, Cognitive" or "Cognitive Impairments" or "Cognitive Impairment" or "Impairment, Cognitive" or "Impairments, Cognitive" or "Mild Cognitive Impairment" or "Cognitive Impairment, Mild" or "Cognitive Impairments, Mild" or "Impairment, Mild Cognitive" or "Impairments, Mild Cognitive" or "Mild Cognitive Impairments" or "Mild Neurocognitive Disorder" or "Disorder, Mild Neurocognitive" or "Disorders, Mild Neurocognitive" or "Mild Neurocognitive Disorders" or "Neurocognitive Disorder, Mild" or "Neurocognitive Disorders, Mild" or "Cognitive Decline" or "Cognitive Declines" or "Decline, Cognitive" or "Declines, Cognitive" or "Mental Deterioration" or "Deterioration, Mental" or "Deteriorations, Mental" or "Mental Deteriorations" or Dementia or Amentia or Amentias or "Senile Paranoid Dementia" or "Dementias, Senile Paranoid" or "Paranoid Dementia, Senile" or "Paranoid Dementias, Senile" or "Senile Paranoid Dementias" or "Familial Dementia" or "Dementia, Familial" or "Dementias, Familial" or Amnesia or Amnesias or "Amnesia-Memory Loss" or "Amnesia Memory Loss" or "Amnesia-Memory Losses" or "Amnesia, Global" or "Amnesias, Global" or "Global Amnesia" or "Global Amnesias" or "Amnestic State" or "Amnestic States" or "State, Amnestic" or "States, Amnestic" or "Amnesia, Tactile" or "Amnesias, Tactile" or "Tactile Amnesia" or "Tactile Amnesias" or "Amnesia, Temporary" or "Amnesias, Temporary" or "Temporary Amnesia" or "Temporary Amnesias" or "Amnesia, Dissociative" or "Amnesias, Dissociative" or "Dissociative Amnesia" or "Dissociative Amnesias" or "Amnesia, Hysterical" or "Amnesias, Hysterical" or "Hysterical Amnesia" or "Hysterical Amnesias" or "Neurodegenerative Diseases" or "Neurodegenerative Disease" or "Degenerative Diseases, Neurologic" or "Neurologic Degenerative Disease" or "Degenerative Neurologic Diseases" or "Degenerative Neurologic Disease" or "Neurologic Disease, Degenerative" or "Neurologic Diseases, Degenerative" or "Nervous System Degenerative Diseases" or "Neurodegenerative Disorders" or "Neurodegenerative Disorder" or "Neurologic Degenerative Conditions" or "Degenerative Condition, Neurologic" or "Degenerative Conditions, Neurologic" or "Neurologic Degenerative Condition" or "Neurologic Degenerative Diseases" or "Degenerative Diseases, Nervous System" or "Degenerative Neurologic Disorders" or "Degenerative Neurologic Disorder" or "Neurologic Disorder, Degenerative" or "Neurologic Disorders, Degenerative" or "Degenerative Diseases, Spinal Cord" or "Degenerative Diseases, Central Nervous System" in Title, Abstract, Keywords in Cochrane Reviews' |
| WEB OF SCIENCE | TOPIC: (Humans) OR TOPIC: ("Man (Taxonomy)") OR TOPIC: ("Modern Man") OR TOPIC: (Human) OR TOPIC: ("Homo sapiens") OR TOPIC: (Adults) OR TOPIC: (Adult) AND TOPIC: ("Masticatory Muscles") OR TOPIC: ("Masticatory Muscle") OR TOPIC: ("Muscle, Masticatory") OR TOPIC: ("Muscles, Masticatory") OR TOPIC: ("Bite Force") OR TOPIC: ("Bite Forces") OR TOPIC: ("Force, Bite") OR TOPIC: ("Forces, Bite") OR TOPIC: ("Occlusal Force") OR TOPIC: ("Force, Occlusal") OR TOPIC: ("Forces, Occlusal") OR TOPIC: ("Occlusal Forces") OR TOPIC: ("Masticatory Force") OR TOPIC: ("Force, Masticatory") OR TOPIC: ("Forces, Masticatory") OR TOPIC: ("Masticatory Forces") OR TOPIC: (Mastication) OR TOPIC: (Chewing) OR TOPIC: (Tooth) OR TOPIC: (Teeth) OR TOPIC: (Dentition) OR TOPIC: (Dentitions) OR TOPIC: ("Tooth loss") OR TOPIC: ("Loss, Tooth") OR TOPIC: ("Mouth, Edentulous") OR TOPIC: ("Edentulous Mouth") OR TOPIC: ("Edentulous Mouths") OR TOPIC: ("Mouth, Toothless") OR TOPIC: ("Toothless Mouth") OR TOPIC: ("Dentition, Permanent") OR TOPIC: ("Permanent Dentition") OR TOPIC: ("Dentition, Secondary") OR TOPIC: ("Secondary Dentition") OR TOPIC: ("Dentition, Adult") OR TOPIC: ("Adult Dentition") AND TOPIC: (”Memory Disorders") OR TOPIC: ("Memory Disorder") OR TOPIC: ("Retention Disorders, Cognitive") OR TOPIC: ("Cognitive Retention Disorder") OR TOPIC: ("Cognitive Retention Disorders") OR TOPIC: ("Retention Disorder, Cognitive") OR TOPIC: ("Memory Loss") OR TOPIC: ("Memory Losses") OR TOPIC: ("Memory Disorder, Semantic") OR TOPIC: ("Memory Disorders, Semantic") OR TOPIC: ("Semantic Memory Disorders") OR TOPIC: ("Semantic Memory Disorder") OR TOPIC: ("Memory Disorder, Spatial") OR TOPIC: ("Memory Disorders, Spatial") OR TOPIC: ("Spatial Memory Disorders") OR TOPIC: ("Spatial Memory Disorder") OR TOPIC: ("Age-Related Memory Disorders") OR TOPIC: ("Age Related Memory Disorders") OR TOPIC: ("Age-Related Memory Disorder") OR TOPIC: ("Memory Disorder, Age-Related") OR TOPIC: ("Memory Disorders, Age-Related") OR TOPIC: ("Memory Disorders, Age Related") OR TOPIC: ("Memory Deficits") OR TOPIC: ("Deficit, Memory") OR TOPIC: ("Deficits, Memory") OR TOPIC: ("Memory Deficit") OR TOPIC: (Cognition) OR TOPIC: (Cognitions) OR TOPIC: ("Cognitive Function") OR TOPIC: ("Cognitive Functions") OR TOPIC: ("Function, Cognitive") OR TOPIC: ("Functions, Cognitive") OR TOPIC: ("Cognition Disorders") OR TOPIC: ("Disorder, Cognition") OR TOPIC: ("Disorders, Cognition") OR TOPIC: ("Overinclusion") OR TOPIC: ("Cognitive Dysfunction") OR TOPIC: ("Cognitive Dysfunctions") OR TOPIC: ("Dysfunction, Cognitive") OR TOPIC: ("Dysfunctions, Cognitive") OR TOPIC: ("Cognitive Impairments") OR TOPIC: ("Cognitive Impairment") OR TOPIC: ("Impairment, Cognitive") OR TOPIC: ("Impairments, Cognitive") OR TOPIC: ("Mild Cognitive Impairment") OR TOPIC: ("Cognitive Impairment, Mild") OR TOPIC: ("Cognitive Impairments, Mild") OR TOPIC: ("Impairment, Mild Cognitive") OR TOPIC: ("Impairments, Mild Cognitive") OR TOPIC: ("Mild Cognitive Impairments”) OR TOPIC: ("Mild Neurocognitive Disorder") OR TOPIC: ("Disorder, Mild Neurocognitive") OR TOPIC: ("Disorders, Mild Neurocognitive") OR TOPIC: ("Mild Neurocognitive Disorders") OR TOPIC: ("Neurocognitive Disorder, Mild") OR TOPIC: ("Neurocognitive Disorders, Mild") OR TOPIC: ("Cognitive Decline") OR TOPIC: ("Cognitive Declines") OR TOPIC: ("Decline, Cognitive") OR TOPIC: ("Declines, Cognitive") OR TOPIC: ("Mental Deterioration") OR TOPIC: ("Deterioration, Mental") OR TOPIC: ("Deteriorations, Mental") OR TOPIC: ("Mental Deteriorations") OR TOPIC: (Dementia) OR TOPIC: (Amentia) OR TOPIC: (Amentias) OR TOPIC: ("Senile Paranoid Dementia") OR TOPIC: ("Dementias, Senile Paranoid") OR TOPIC: ("Paranoid Dementia, Senile") OR TOPIC: ("Paranoid Dementias, Senile") OR TOPIC: ("Senile Paranoid Dementias") OR TOPIC: ("Familial Dementia") OR TOPIC: ("Dementia, Familial") OR TOPIC: ("Dementias, Familial") OR TOPIC: (Amnesia) OR TOPIC: (Amnesias) OR TOPIC: ("Amnesia-Memory Loss") OR TOPIC: ("Amnesia Memory Loss") OR TOPIC: ("Amnesia-Memory Losses") OR TOPIC: ("Amnesia, Global") OR TOPIC: ("Amnesias, Global") OR TOPIC: ("Global Amnesia") OR TOPIC: ("Global Amnesias") OR TOPIC: ("Amnestic State") OR TOPIC: ("Amnestic States") OR TOPIC: ("State, Amnestic") OR TOPIC: ("States, Amnestic") OR TOPIC: ("Amnesia, Tactile") OR TOPIC: ("Amnesias, Tactile") OR TOPIC: ("Tactile Amnesia") OR TOPIC: ("Tactile Amnesias") OR TOPIC: ("Amnesia, Temporary") OR TOPIC: ("Amnesias, Temporary") OR TOPIC: ("Temporary Amnesia") OR TOPIC: ("Temporary Amnesias") OR TOPIC: ("Amnesia, Dissociative") OR TOPIC: ("Amnesias, Dissociative") OR TOPIC: ("Dissociative Amnesia") OR TOPIC: ("Dissociative Amnesias") OR TOPIC: ("Amnesia, Hysterical") OR TOPIC: ("Neurodegenerative Disease") OR TOPIC: ("Degenerative Diseases, Neurologic") OR TOPIC: ("Neurologic Degenerative Disease") OR TOPIC: ("Degenerative Neurologic Diseases") OR TOPIC: ("Degenerative Neurologic Disease") OR TOPIC: ("Neurologic Disease, Degenerative") OR TOPIC: ("Neurologic Diseases, Degenerative") OR TOPIC: ("Nervous System Degenerative Diseases") OR TOPIC: ("Neurodegenerative Disorders") OR TOPIC: ("Neurodegenerative Disorder") OR TOPIC: ("Neurologic Degenerative Conditions") OR TOPIC: ("Degenerative Condition, Neurologic") OR TOPIC: ("Degenerative Conditions, Neurologic") OR TOPIC: ("Neurologic Degenerative Condition") OR TOPIC: ("Neurologic Degenerative Diseases") OR TOPIC: ("Degenerative Diseases, Nervous System") OR TOPIC: ("Degenerative Neurologic Disorders") OR TOPIC: ("Degenerative Neurologic Disorder") OR TOPIC: ("Neurologic Disorder, Degenerative") OR TOPIC: ("Neurologic Disorders, Degenerative") OR TOPIC: ("Degenerative Diseases, Spinal Cord") OR TOPIC: ("Degenerative Diseases, Central Nervous System”) |
| GOOGLE SCHOLAR | Humans OR Adults OR Tooth OR Dentition OR “Tooth loss” OR “Mouth, Edentulous” OR “Dentition, Permanent” OR “Masticatory Muscles” OR “Bite Force” OR Mastication OR “Memory Disorders” OR Cognition OR “Cognition Disorders” OR “Cognitive Dysfunction” OR Dementia OR Amnesia OR “Neurodegenerative Diseases” |

1. **Criteria for quality assessment**

| **Guideline** | **Checklist** | **Description** |
| --- | --- | --- |
| Study design apropriate to objectives? | Objective common design |  |
|  | Prevalence Cross-sectional |  |
|  | Prognosis Cohort |  |
|  | Treatment Controled trial |  |
|  | Cause Cohort, case-control, cross-sectional |  |
| Study sample representative? | Source of sample | As for Study sample representative: In the topic "Source of sample" (0) in cases of detailed origin, (+) specifies the origin of only one group and (++) does not specify the origin of the groups. |
|  | Sampling method | In the item "Sampling method" was assigned (0) randomization, (+) randomization of one of the groups and (++) without randomization of the groups. |
|  | Sample size | For ¨Sample size¨ a minor problem (+) was considered when the study did not the representativity of the sample was clear or did not report sample calculations. For a larger problem (++) absence of sample calculation and number of participants less than 50 participants, (0) absence of the factors mentioned above. |
|  | Entry criteria/exclusion | Regarding the "Entry criteria / exclusion", a minor problem was attributed (+) when demographics factors, lifestyle, education, age, health conditions, were not included as covariates. In the case of more than one item previously mentioned, considered a major problem (++). |
|  | Non-respondents | For "Non-respondents" (0) there was no refusal, all participated in the study, (+) there was refusal, but did not compromise the sample and (++) there was refusal and impairment of the sample size. |
| Control group acceptable? | Definition of controls | In this domain (0) when describing all the characteristics of the control group, (+) leaves some pending information such as, the origin of the control group, the selection criteria of the group (++) when two or more of the previous items are present. |
|  | Source of controls | In this item was considered (0) when referring to the control group, (+) the origin of the groups is different, but it justifies the reason for such difference and (++) the origin of the groups are different and does not justify why. |
|  | Matching/randomization | For "Matching / randomization" (0) when pairing between groups, (+) there is no description of how much to the randomization, nevertheless they do pairing of the groups and (++) there is no description of as far as the randomization and do not make pairing of the groups. |
|  | Comparable characteristics | For "Comparable characteristics" we considered (0) paired or non-paired groups matched by the impossibility of being adjusted later and (++) the presence of variables that were neither matched nor adjusted. |
| Quality of measurements and outcomes? | Validity | For this domain, it was considered (0) when the method of evaluation of the cognitive pattern applied is adequate and widely used; (+) when using a questionable or unvalidated evaluation method, but has adequate specificity with good sensitivity; (++) when using an unvalidated method, and it does not have adequate specificity and does not present good sensitivity. |
|  | Reproducibility | Regarding "Reproducibility" it was considered (0) whether the evaluation methods were well described, which can be reproduced; (+) if there was no description of the evaluation of cognition, evaluations at different times, application of different methods to evaluate the groups; (++) when two or more of the previous items are present. |
|  | Blindness | Regarding this, the condition of the study participants was considered "Blind", in this case being assigned the signal (0), in cases of "not blind" the signal (++). |
|  | Quality control | For "quality control", they were considered as a problem when evaluators were not calibrated; the application of cognitive evaluation tests or tooth loss measurement by untrained professionals or self-reported by individuals; when two of these characteristics were present it was considered as a problem (+) and major problems (++) if more than two of these characteristics are gifts. |
| Completeness | Compliance | For "Compliance" (0) the sample size remains from the beginning to the end or decreases without compromising the power of the test; (+) difference in the sample size at the end of the study, compromising the power of the however justifies and adjusts; (++) difference in sample size at the end of the study, compromising the power of the test, does not justify and does not adjust. |
|  | Drop outs | Dropouts (0) when there are no losses during the study (+) when there is withdrawal that compromises the inclusion criteria, such as age, sex; (++) when there is withdrawal and compromises more than one criterion. |
|  | Deaths |  |
|  | Missing data | For "Missing data" (0) no there was a loss of data; (+) there was loss of sample data without compromising the analysis final statistics; (++) there was loss of data of the sample compromising the analysis final statistic. |
| Distorting influences? | Extraneous treatments | Was considered (0) when there are no external influences; (+) when there are external influences, but that does not interfere in the results; (++) when there are external influences and interferes with the results. |
|  | Contamination | For "Contamination" was attributed NA because they are observational studies. |
|  | Changes over time | (0) data collected in the same period of time; (+) data collected from the control group and the study group at different times may cause distortion; (++) the previous item associated with data from studies already published. |
|  | Confounding factors | As for the "Confounding factors”, age, sex, health conditions well known associated to cognitive deficit, demographic and social lifestyle, weren’t adjusted; A "minor" (+) problem was attributed when 1 or 2 of these characteristics were present and a "larger" problem; (++) if there were 3 or more. |
|  | Distortion reduced by analysis | In the item "Distortion reduced by analysis", it was considered (0) when adjustments of covariables that present distortions; (+) cites the adjustment, but does not say the criteria; (++) presents covariables with distortions and does not make any type of adjustment. |

1. **Reasons for exclusion**

| **Reference** | **Reason for exclusion** |
| --- | --- |
| Bergdahl, M., Habib, R., Bergdahl, J., Nyberg, L., & NILSSON, L. G. (2007). Natural teeth and cognitive function in humans. *Scandinavian journal of psychology*, *48*(6), 557-565. | Dental status analysis method unable to assess masticatory dysfunction accordingly our eligibility criteria |
| Bergdahl, M., Habib, R., Bergdahl, J., Nyberg, L., & NILSSON, L. G. (2007). Natural teeth and cognitive function in humans. *Scandinavian journal of psychology*, *48*(6), 557-565. | Dental status analysis method unable to assess masticatory dysfunction accordingly our eligibility criteria |
| Chen, X., Shuman, S. K., Hodges, J. S., Gatewood, L. C., & Xu, J. (2010). Patterns of tooth loss in older adults with and without dementia: a retrospective study based on a Minnesota cohort. *Journal of the American Geriatrics Society*, *58*(12), 2300-2307. | Dental status analysis method unable to assess masticatory dysfunction accordingly our eligibility criteria |
| Del Brutto, O. H., Gardener, H., Del Brutto, V. J., Maestre, G. E., Zambrano, M., Montenegro, J. E., & Wright, C. B. (2014). Edentulism associates with worse cognitive performance in community-dwelling elders in rural Ecuador: results of the Atahualpa project. *Journal of community health*, *39*(6), 1097-1100. | Dental status analysis method unable to assess masticatory dysfunction accordingly our eligibility criteria |
| Dintica, C. S., Rizzuto, D., Marseglia, A., Kalpouzos, G., Welmer, A. K., Wårdh, I., ... & Xu, W. (2018). Tooth loss is associated with accelerated cognitive decline and volumetric brain differences: a population-based study. *Neurobiology of aging*, *67*, 23-30. | Dental status analysis method unable to assess masticatory dysfunction accordingly our eligibility criteria |
| Kaye, E. K., Valencia, A., Baba, N., Spiro III, A., Dietrich, T., & Garcia, R. I. (2010). Tooth loss and periodontal disease predict poor cognitive function in older men. *Journal of the American Geriatrics Society*, *58*(4), 713-718. | Dental status analysis method unable to assess masticatory dysfunction accordingly our eligibility criteria |
| Grabe, H. J., Schwahn, C., Völzke, H., Spitzer, C., Freyberger, H. J., John, U., ... & Kocher, T. (2009). Tooth loss and cognitive impairment. *Journal of clinical periodontology*, *36*(7), 550-557. | Dental status analysis method unable to assess masticatory dysfunction accordingly our eligibility criteria |
| Ikebe, K., Gondo, Y., Kamide, K., Masui, Y., Ishizaki, T., Arai, Y., ... & Okubo, H. (2018). Occlusal force is correlated with cognitive function directly as well as indirectly via food intake in community-dwelling older Japanese: From the SONIC study. *PloS one*, *13*(1), e0190741. | Dental status analysis method unable to assess masticatory dysfunction accordingly our eligibility criteria |
| Kim, E. K., Lee, S. K., Choi, Y. H., Tanaka, M., Hirotsu, K., Kim, H. C., ... & Amano, A. (2017). Relationship between chewing ability and cognitive impairment in the rural elderly. *Archives of gerontology and geriatrics*, *70*, 209-213. | Absence of control group |
| Luo, J., Wu, B., Zhao, Q., Guo, Q., Meng, H., Yu, L., ... & Ding, D. (2015). Association between tooth loss and cognitive function among 3063 Chinese older adults: a community-based study. *PloS one*, *10*(3), e0120986. | Dental status analysis method unable to assess masticatory dysfunction accordingly our eligibility criteria |
| Lexomboon, D., Trulsson, M., Wårdh, I., & Parker, M. G. (2012). Chewing ability and tooth loss: association with cognitive impairment in an elderly population study. *Journal of the American Geriatrics Society*, *60*(10), 1951-1956. | Dental status analysis method unable to assess masticatory dysfunction accordingly our eligibility criteria |
| Miura, H., Yamasaki, K., Kariyasu, M., Miura, K., & Sumi, Y. (2003). Relationship between cognitive function and mastication in elderly females. *Journal of oral rehabilitation*, *30*(8), 808-811. | Dental status analysis method unable to assess masticatory dysfunction accordingly our eligibility criteria |
| Okamoto, N., Morikawa, M., Yanagi, M., Amano, N., Tomioka, K., Hazaki, K., ... & Kurumatani, N. (2015). Association of tooth loss with development of swallowing problems in community-dwelling independent elderly population: the Fujiwara-kyo study. *Journals of Gerontology Series A: Biomedical Sciences and Medical Sciences*, *70*(12), 1548-1554. | Dental status analysis method unable to assess masticatory dysfunction accordingly our eligibility criteria |
| Okamoto, N., Morikawa, M., Tomioka, K., Yanagi, M., Amano, N., & Kurumatani, N. (2015). Association between tooth loss and the development of mild memory impairment in the elderly: the Fujiwara-kyo Study. *Journal of Alzheimer's Disease*, *44*(3), 777-786. | Dental status analysis method unable to assess masticatory dysfunction accordingly our eligibility criteria |
| Okamoto, N., Morikawa, M., Amano, N., Yanagi, M., Takasawa, S., & Kurumatani, N. (2017). Effects of Tooth Loss and the Apolipoprotein E ɛ4 Allele on Mild Memory Impairment in the Fujiwara-kyo Study of Japan: A Nested Case-Control Study. *Journal of Alzheimer's Disease*, *55*(2), 575-583. | Dental status analysis method unable to assess masticatory dysfunction accordingly our eligibility criteria |
| Stein, P. S., Desrosiers, M., Donegan, S. J., Yepes, J. F., & Kryscio, R. J. (2007). Tooth loss, dementia and neuropathology in the Nun study. *The Journal of the American Dental Association*, *138*(10), 1314-1322. | Patients pre-diagnosed with dementia |
| Paganini‐Hill, A., White, S. C., & Atchison, K. A. (2012). Dentition, Dental Health Habits, and Dementia: The L eisure W orld C ohort S tudy. *Journal of the American Geriatrics Society*, *60*(8), 1556-1563. | Dental status analysis method unable to assess masticatory dysfunction accordingly our eligibility criteria |
| Takata, Y., Ansai, T., Soh, I., Sonoki, K., Awano, S., Hamasaki, T., ... & Takehara, T. (2009). Cognitive function and number of teeth in a community‐dwelling elderly population without dementia. *Journal of Oral Rehabilitation*, *36*(11), 808-813. | Dental status analysis method unable to assess masticatory dysfunction accordingly our eligibility criteria |
| Peres, M. A., Bastos, J. L., Watt, R. G., Xavier, A. J., Barbato, P. R., & D’Orsi, E. (2015). Tooth loss is associated with severe cognitive impairment among older people: findings from a population-based study in Brazil. *Aging & mental health*, *19*(10), 876-884. | Dental status analysis method unable to assess masticatory dysfunction accordingly our eligibility criteria |
| Paganini‐Hill, A., White, S. C., & Atchison, K. A. (2012). Dentition, Dental Health Habits, and Dementia: The L eisure W orld C ohort S tudy. *Journal of the American Geriatrics Society*, *60*(8), 1556-1563. | Dental status analysis method unable to assess masticatory dysfunction accordingly our eligibility criteria |
| Taraghi, Z., Fanni-Saberi, L., Yazdani-Charati, J., & Meskini, L. (2017). The Relationship Between Oral Health and Cognitive Status of the Elderly. *Iranian Red Crescent Medical Journal*, *19*(9). | Dental status analysis method unable to assess masticatory dysfunction accordingly our eligibility criteria |
| Tsakos, G., Watt, R. G., Rouxel, P. L., de Oliveira, C., & Demakakos, P. (2015). Tooth loss associated with physical and cognitive decline in older adults. *Journal of the American Geriatrics Society*, *63*(1), 91-99. | Dental status analysis method unable to assess masticatory dysfunction accordingly our eligibility criteria |

1. **Funnel plot**

**
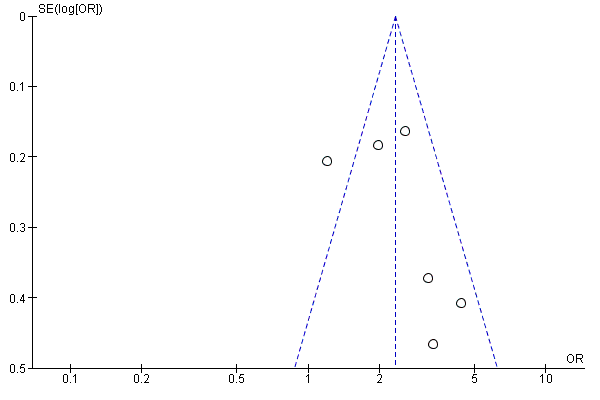
**
